# GREPore-seq: A Robust Workflow to Detect Changes After Gene Editing Through Long-range PCR and Nanopore Sequencing

**DOI:** 10.1016/j.gpb.2022.06.002

**Published:** 2022-06-23

**Authors:** Zi-Jun Quan, Si-Ang Li, Zhi-Xue Yang, Juan-Juan Zhao, Guo-Hua Li, Feng Zhang, Wei Wen, Tao Cheng, Xiao-Bing Zhang

**Affiliations:** 1 State Key Laboratory of Experimental Hematology, National Clinical Research Center for Blood Diseases, Haihe Laboratory of Cell Ecosystem, Institute of Hematology & Blood Diseases Hospital, Chinese Academy of Medical Sciences & Peking Union Medical College, Tianjin 300020, China; 2 Center for Stem Cell Medicine, Chinese Academy of Medical Sciences, Tianjin 300020, China; 3 Department of Stem Cell & Regenerative Medicine, Peking Union Medical College, Tianjin 300020, China

**Keywords:** CRISPR/Cas9, Genetic change, Long-range PCR, Nanopore sequencing, GREPore-seq

## Abstract

To achieve the enormous potential of gene-editing technology in clinical therapies, one needs to evaluate both the on-target efficiency and unintended editing consequences comprehensively. However, there is a lack of a pipelined, large-scale, and economical workflow for detecting genome editing outcomes, in particular insertion or deletion of a large fragment. Here, we describe an approach for efficient and accurate detection of multiple **genetic changes** after **CRISPR/Cas9** editing by pooled **nanopore sequencing** of barcoded **long-range PCR** products. Recognizing the high error rates of Oxford nanopore sequencing, we developed a novel pipeline to capture the barcoded sequences by grepping reads of nanopore amplicon sequencing (**GREPore-seq**). GREPore-seq can assess nonhomologous end-joining (NHEJ)-mediated double-stranded oligodeoxynucleotide (dsODN) insertions with comparable accuracy to Illumina next-generation sequencing (NGS). GREPore-seq also reveals a full spectrum of homology-directed repair (HDR)-mediated large gene knock-in, correlating well with the fluorescence-activated cell sorting (FACS) analysis results. Of note, we discovered low-level fragmented and full-length plasmid backbone insertion at the CRISPR cutting site. Therefore, we have established a practical workflow to evaluate various genetic changes, including quantifying insertions of short dsODNs, knock-ins of long pieces, plasmid insertions, and large fragment deletions after CRISPR/Cas9-mediated editing. GREPore-seq is freely available at GitHub (https://github.com/lisiang/GREPore-seq) and the National Genomics Data Center (NGDC) BioCode (https://ngdc.cncb.ac.cn/biocode/tools/BT007293).

## Introduction

The RNA-guided clustered, regularly interspaced, short palindromic repeats (CRISPR)/CRISPR-associated (Cas) DNA endonuclease system has been harnessed for genome editing [1]. The genetic changes after CRISPR/Cas9 editing in humans have been extensively investigated. Generally, the repair of DNA double-strand breaks (DSBs) after CRISPR editing induces gene knockout mediated by nonhomologous end-joining (NHEJ) and precise gene correction by homology-directed repair (HDR) [2], [3], [4]. However, several researchers recently identified unintended large fragment deletions (kilobase scale) and even complex genomic rearrangements at target sites of gene-edited cells and human embryos [5], [6], [7], [8], [9], [10]. Due to the potential clinical applications of CRISPR/Cas9, it is imperative to assess genome editing outcomes [11], [12] comprehensively.

In recent years, next-generation sequencing (NGS) has been widely used to assess NHEJ-mediated indels or HDR-mediated small changes due to its high-throughput capacity and low error rate. The NGS data can be analyzed with CRISPResso2 to determine the editing patterns and outcomes [13], [14]. However, NGS technologies are limited by their short read length, usually paired ends of 150 bp, making it impossible to accurately detect large fragment knock-in mediated by the HDR pathway or large deletions after DSBs. The advent of the third-generation sequencing (3GS) technologies ushered in an era of long-length reads, breaking the bottleneck of NGS technologies. These technologies directly read single molecules, enabling real-time sequencing and increasing read length to tens of thousands of bases per read [15], [16]. The most widely used 3GS platforms are Pacific Biosciences (PacBio) and Oxford Nanopore Technologies (ONT). Unlike PacBio platform sequencing by synthesis (SBS), ONT detects DNA bases by monitoring the variation in electric currents while a stretch of nucleotides crosses a nanopore. Nanopore sequencing commercialized by ONT can produce ultralong reads exceeding a mega‐base and is less likely to have inherent limitations in potential read length, as it is not based on SBS. With its affordability, portability, and speed in data production, ONT has been used to detect large insertions or deletions after gene editing [17], [18], [19], [20].

Amplicon sequencing entails amplifying the target sequence by polymerase chain reaction (PCR). However, amplifying kilobases from genomic DNA (gDNA) is more challenging than PCR of short amplicons. In 1992, the use of new PCR conditions allowed for amplification of up to 5 kb [21]. More recently, novel polymerases increased the size of amplicons to over 30 kb [22]. Coupled with PacBio sequencing [6], [9], these advances in PCR make it feasible to identify large insertions and deletions (indels) in genomic regions of interest. However, PacBio is less attractive than ONT in read length, portability, and cost. As such, we elected the ONT platform for long amplicon sequencing and data analysis pipeline development.

However, nanopore sequencing has a high systematic error rate compared to NGS [19], [20]; the previously developed toolkits are not applicable for the analysis of 3GS data. Therefore, we attempted to create a grepping pooled nanopore sequencing workflow, grepping reads of nanopore amplicon sequencing (GREPore-seq), after considering the strengths and intrinsic limitations of 3GS. GREPore-seq combines indel-correcting DNA barcodes [23] with the sequencing of long amplicons on the ONT platforms. As a proof of concept, we can accurately detect genetic changes such as NHEJ-mediated double-stranded oligodeoxynucleotide (dsODN) insertions, HDR knock-ins, large deletions after CRISPR/Cas9 editing, and accidental insertions of plasmid backbone (BB) at the cutting site. This robust workflow is characterized by multiple features: (1) ease of implementation in any computer; (2) ability to simultaneously analyze pools of amplicons tagged with dozens, even hundreds of barcodes; (3) economy of scale; (4) high-level data retrieval; and (5) low false discovery rate (FDR).

## Results

### Efficient extraction of long-range PCR reads from nanopore data

**Figure 1:**
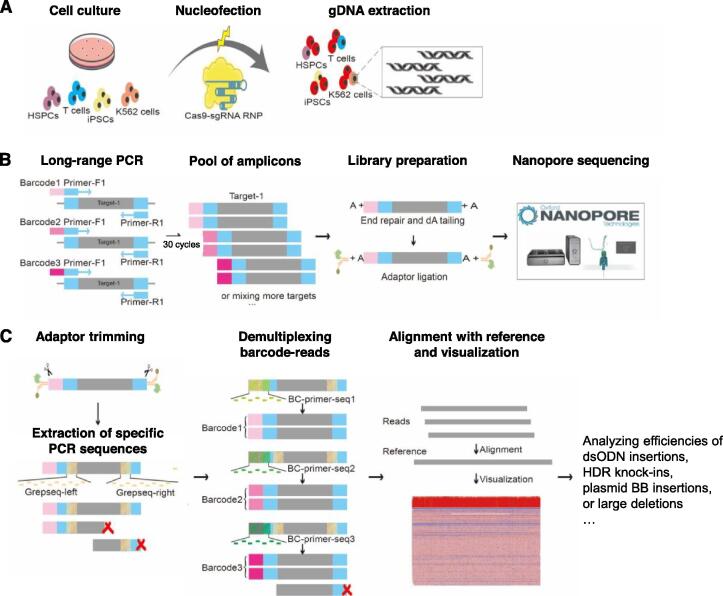
**A schematic overview of GREPore-seq workflow** **A.** Step 1, laboratory process of cell culture, nucleofection, and gDNA extraction. **B.** Step 2, amplicon library preparation and nanopore sequencing. **C.** Step 3, GREPore-seq bioinformatic analysis. gDNA, genomic DNA; HSPC, hematopoietic stem and progenitor cell; RNP, ribonucleoprotein; sgRNA, single guide RNA; GREPore-seq, grepping reads of nanopore amplicon sequencing; dsODN, double-stranded oligodeoxynucleotide; HDR, homology-directed repair; BB, backbone.

We designed and extensively optimized a GREPore-seq protocol to identify significant genetic changes after CRISPR-mediated dsDNA cleavage and NHEJ- or HDR-mediated editing, as illustrated in Figure 1. First, K562 cells, human T cells, hematopoietic stem and progenitor cells (HSPCs), or induced pluripotent stem cells (iPSCs) were nucleofected with ribonucleoprotein (RNP) for editing. Three to four days later, we extracted gDNA and performed long-range PCR targeting 4–8 kb surrounding the guide RNA (gRNA) on-target sites. We tagged the forward primers with indel-correcting DNA barcodes at the 5′-end to enable pooled sequencing of long amplicons [23]. Amplicons with distinct barcodes were pooled for nanopore sequencing (Figure 1A and B). After acquiring the raw data that were processed with Guppy [24], the adaptor was trimmed by Porechop [25], and reads were initially binned based on the two terminal Grepseqs of specific PCR products. Subsequently, reads were demultiplexed using BC-primer-seq of barcodes. The demultiplexed fastq data were then aligned with reference amplicon sequences (Table S1) using Minimap2 [26]. Finally, the sorted bam files were visualized with integrative genomics viewer (IGV) [27], [28]. We also developed scripts to analyze dsODN insertions, HDR knock-ins, plasmid BB insertions, or large deletions after gene editing (Figure 1C).

**Figure 2:**
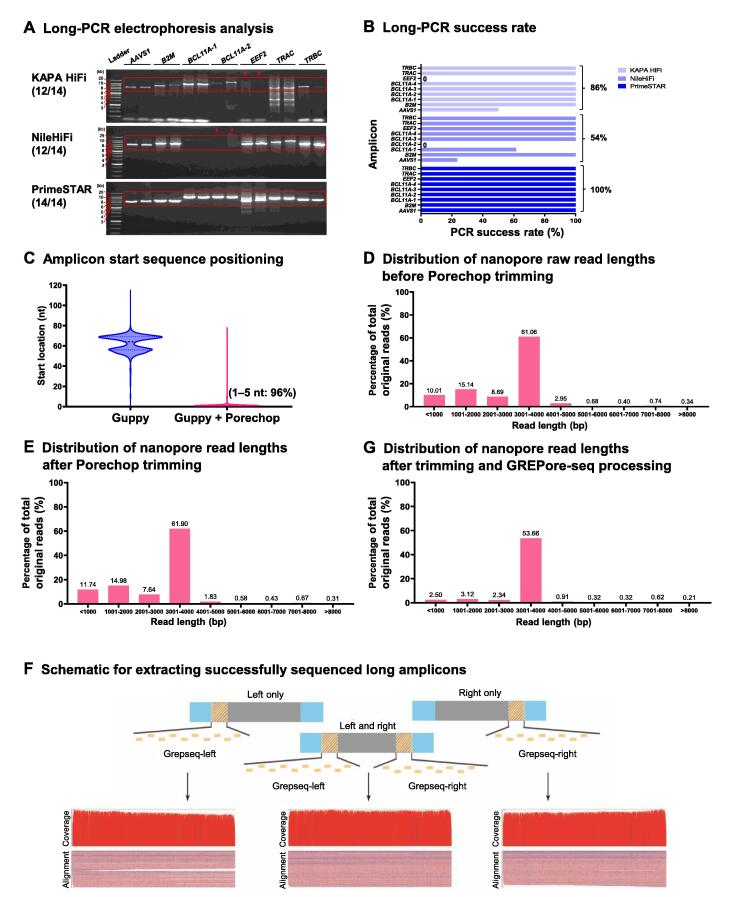
**Successful long-range PCR, trimming, and retrieval of full-length amplicon reads** **A.** Comparison of three DNA polymerase kits. The quality and quantity of PCR products were assessed by electrophoresis on agarose gels. Specific primers (without barcode) for seven sites were used to amplify WT targets, each with two technical replicates. Red cross indicates failed amplification; red frames indicate expected products (*AAVS1*, 3928 bp; *B2M*, 5666 bp; *BCL11A-1*, 8159 bp; *BCL11A-2*, 8443 bp; *EEF2*, 5287 bp; *TRAC*, 6485 bp; *TRBC*, 5093 bp). **B.** PCR success rates of PrimeSTAR, KAPA HiFi, and NileHiFi among 135 reactions. **C.** Removal of adaptors with Porechop. We used the command “seqkit locate -p” and the barcode “AACGGACT” (for *BCL11A-3* primer-F) to detect the start location after Guppy or Porechop trimming. The two peaks in blue indicate nanopore sequencing adaptors. **D.** Distribution of nanopore raw read lengths before Porechop trimming. **E.** Distribution of nanopore read lengths after Porechop trimming. The percentages of read numbers were normalized to raw reads before Porechop processing. **F.** Strategy for retrieving full-length amplicon reads. A schematic of the Grepseq-left and Grepseq-right generations is shown. The retrieved *BCL11A-3* amplicon reads were visualized with IGV after sampling 200 reads using the command “seqkit sample 200”. **G.** Distribution of nanopore read lengths after extracting the *BCL11A-3* PCR product (3863 bp) by GERPore-seq. The read numbers were displayed as percentage of raw reads before trimming. WT, wild-type.

First, we optimized PCR conditions by comparing three DNA polymerase kits commercialized for long-range PCR amplification, including KAPA HiFi DNA polymerase (Kapa Biosystems), NileHiFi long amplicon PCR kit (GeneCopoeia), and PrimeSTAR GXL DNA polymerase (Takara Bio). We PCR-amplified various gDNA target regions in the head-to-head comparison, ranging from 4 to 8 kb at *AAVS1*, *B2M*, *EEF2*, *TRAC*, *TRBC*, and two *BCL11A* loci (*BCL11A-1* and *BCL11A-2*) of human primary T cells or iPSCs. The quality and quantity of PCR products were assessed by electrophoresis on agarose gels (Figure 2A). KAPA HiFi performed similarly to NileHiFi, both of which failed to amplify 2 of 14 PCR products (Figure 2A). In comparison, PrimeSTAR succeeded in amplifying all these products. Moreover, NileHiFi fell short in amplifying the *BCL11A-1* and *BCL11A-2* products as long as 8 kb. We also observed a bright, single band of expected size on most products using PrimeSTAR. In contrast, KAPA HiFi gave a lower yield and more primer dimers, indicating PrimeSTAR’s superior specificity and productivity. Among a total of 135 reactions, the success rates of PrimeSTAR, KAPA HiFi, and NileHiFi were 100%, 86%, and 54%, respectively (Figure 2B). Therefore, PrimeSTAR was used for long-range PCR in subsequent experiments.

A pool of dozens of long amplicons was sequenced on PromethION. To develop a new approach for retrieving each amplicon data, we first compared the tools for trimming sequencing adaptors. Guppy is a neural network-based basecaller that also performs clipping of nanopore adaptors. However, as exemplified by the *BCL11A-3* amplicon data, we found that the first bases of expected amplicons started at a 40–80-nucleotide (nt) location from the beginning of the reads when trimmed by Guppy alone. In addition, we observed two peaks, possibly indicating nanopore sequencing adaptors at both ends. We then adopted Porechop for further trimming, which successfully trimmed 96% of reads, leaving a 1–5-nt residual adaptor sequence (Figure 2C). These results demonstrate that Guppy combined with Porechop is optimal for data trimming.

We then analyzed the distribution of nanopore read lengths before and after Porechop trimming in a batch containing only one type of PCR product, *BCL11A-3*, whose expected length was 3863 bp. We found that it included reads of the expected length and longer and shorter reads (Figure 2D and E), which could not be eliminated by Porechop trimming. We interpreted these data as artifacts of nanopore sequencing since the PCR products were specific and identical in length. A straightforward strategy is directly filtering out too long or too short reads, an algorithm used by quality filtering software such as Filtlong (https://github.com/rrwick/Filtlong) and Nanofilt [29]. However, these approaches will erroneously deplete reads with large indels. This scenario motivated us to develop an effective scheme with a minimal FDR.

Considering the systematic error of short indels in nanopore sequencing [20], we designed a potentially less sensitive approach to short indels. First, we generated multiple lines of overlapping reference amplicon sequences to capture as many target reads as possible. Specifically, we made a string of *k*-mers with a length of 15 nt and a step of 5 nt in the range of 20–90 nt at both ends of the trimmed sequences, which we named Grepseq-left and Grepseq-right. We then compared data extraction using Grepseq-left and/or Grepseq-right from *BCL11A-3* wild-type (WT) sequences. Sequence mapping with Minimap2 and visualization with IGV showed distinct patterns of the three data processing schemes (Figure 2F). As expected, using a single Grepseq for read capture led to the retrieval of incomplete sequences, an ONT artifact likely due to transient nanopore blockage. However, we significantly enriched the almost perfect reads when utilizing both left and right Grepseqs. Analysis of the distribution of read lengths showed that 54% of reads were near the expected size (3863 bp), the longer reads were reduced by 53% (from ∼ 5% to ∼ 2%), and the shorter reads were decreased by 76% (from ∼ 34% to ∼ 8%) after extracting full-length amplicon reads by GREPore-seq (Figure 2G). As such, we incorporated this retrieval strategy in the GREPore-seq pipeline. Together, we developed GREPore-seq to extract reads of the target amplicons effectively.

### GREPore-seq effectively demultiplexes barcoded nanopore amplicon reads

**Figure 3:**
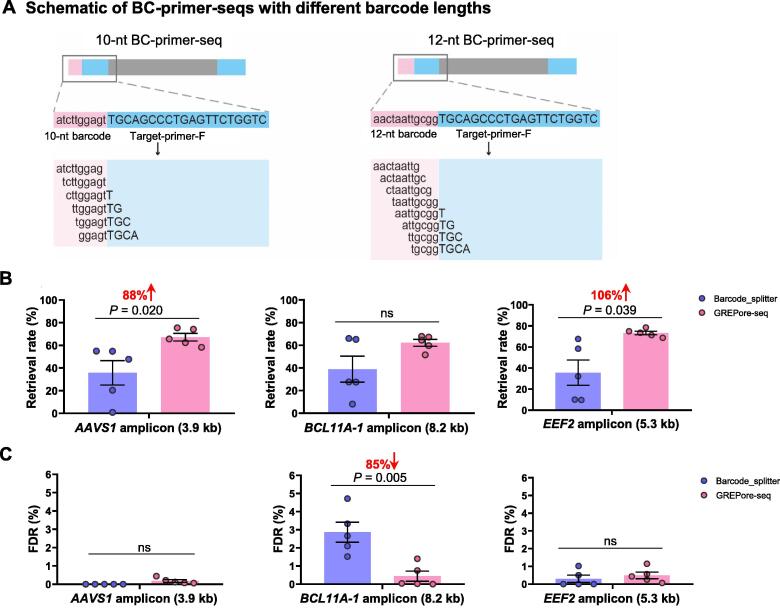
**GREPore-seq effectively retrieves amplicon-specific nanopore reads with barcodes** **A.** A schematic overview of the BC-primer-seqs with 10-nt or 12-nt barcodes. BC-primer-seqs are stretches of short overlapping sequences for data retrieval. **B.** and **C.** Higher data retrieval rate (B) and lower FDR (C) of GREPore-seq compared with those of Barcode_splitter.Data are represented as mean ± SEM (*n* = 5 independent experiments). Paired two-sided Student’s *t*-tests were conducted. “ns” means no significance (*P* > 0.05). FDR, false discovery rate.

To avoid batch discrepancies and reduce the costs of nanopore sequencing, we performed long-range PCR with a customized set of tailed primers, including a barcode sequence on the forward primer and a reverse primer. Of note, PCR amplification was not affected after tagging a barcode of 10, 12, or 14 nt on the forward primer (details not shown). After extracting sequenced amplicon data, reads were demultiplexed using BC-primer-seq. It was a string of *k*-mers with a length of 9 nt and a step of 1 nt, consisting of barcode and forward primer, which contains at least the last 4 nt of barcode regardless of different barcode lengths (Figure 3A). As Barcode_splitter (barcode-splitter · PyPI) is widely used to demultiplex NGS data, we compared GREPore-seq with Barcode_splitter on processing nanopore sequencing data in batches containing *AAVS1*, *BCL11A-3*, and *EEF2* amplicons.

We merged all the demultiplexing files and removed duplicated reads using the ‘seqkit rmdup’ command [30] to determine the demultiplexing retrieval rate and FDR. Demultiplexing retrieval rate was defined as ratios of reads before and after extracting the amplicon-specific reads by Grepseqs. FDR was defined by the proportion of duplicated reads. We observed that GREPore-seq recovered greater quantities of demultiplexed data for all amplicons, ranging from 60% to 106%, with a significant difference at *AAVS1* (*P* = 0.020) and *EEF2* (*P* = 0.039) relative to Barcode_splitter (Figure 3B). In addition, GREPore-seq showed a significant reduction in FDR at *BCL11A-1* (85%, *P* = 0.005) and maintained low FDR at *AAVS1* and *EEF2* (Figure 3C). Therefore, these data demonstrate that GREPore-seq performs better than Barcode_splitter in demultiplexing barcoded long amplicons after nanopore sequencing.

### Strategy for retrieving full-length amplicon-specific nanopore data

**Figure 4:**
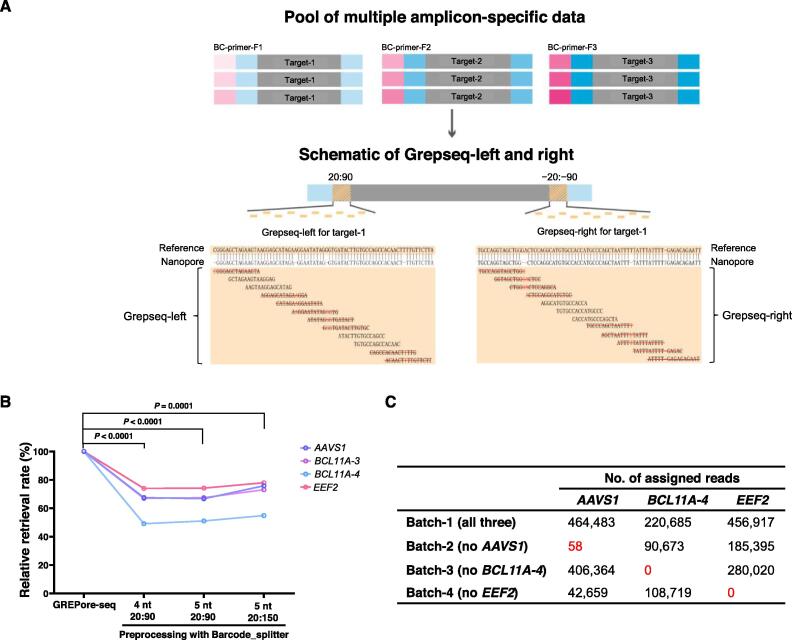
**Strategy for retrieving full-length nanopore amplicon reads** **A.** A schematic overview of Grepseqs and extraction for three distinct target reads. Bases and “–” marked in red indicate the mismatches aligned between the reference and a nanopore read. **B.** Pre-processing with Barcode_splitter decreases the data retrieval rate. Data were normalized to processing with GREPore-seq alone. The sequence of 4 nt or 5 nt at the beginning was used in Barcode_splitter. 20:90 or 20:150 represents the range of Grepseqs used for amplicon-specific read extraction. The data were statistically analyzed by two-way ANOVA (adjusted *P* values were indicated). **C.** Low misassignment of amplicon reads by GREPore-seq. Numbers in red indicate erroneously assigned reads.

One needs to pool multiple unique site amplicons in a single sequencing specimen to achieve cost-effectiveness. The GREPore-seq pipeline includes a module to separate amplicon-specific reads into individual bins. Figure 4A shows a schematic overview of Grepseqs and extraction for three distinct target reads. Since GREPore-seq requires intensive computation, we asked if Barcode_splitter could pre-extract amplicon-specific data with a more significant retrieval rate and higher speed than GREPore-seq alone. For this purpose, we used 4-nt or 5-nt bases and allowed two mismatches for Barcode_splitter analysis. Unfortunately, pre-processing with Barcode_splitter using three sets of parameters showed significantly lower (20%–50%) data recovery than GREPore-seq analysis alone (Figure 4B). Therefore, we discontinued using this approach.

We then assessed the FDR of extracting amplicon-specific data. The control dataset (batch-1) contains sequencing data of three amplicons, *AAVS1*, *BCL11A-4* (similar to *BCL11A-1* but with a different length), and *EEF2*, while reads of one locus were omitted in the three test datasets (batch-2 to batch-4). We then used GREPore-seq to retrieve all three amplicon-specific reads. In datasets without *BCL11A-4* or *EEF2* reads, no misassignment was observed. In comparison, 58 reads were erroneously assigned to the *AAVS1* amplicon in another dataset. However, given the 1,575,888 reads in this dataset, the FDR was lower than 0.01%. Therefore, GREPore-seq correctly bins different amplicon reads with acceptable FDR.

### GREPore-seq correctly identifies short dsODN insertions

**Figure 5:**
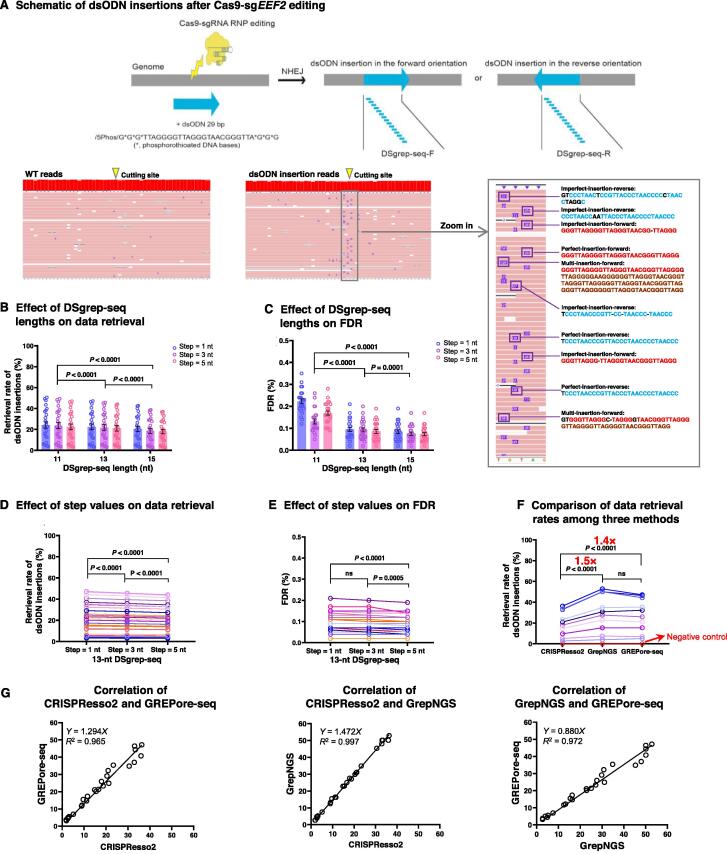
**GREPore-seq correctly identifies short dsODN insertions** **A.** Top: A schematic overview of 29-bp dsODN insertions at CRISPR/Cas9-mediated DSBs and generation of DSgrep-seqs for retrieving reads with the insertion. Bottom: Visualization of WT- or dsODN-inserted-amplicon reads and showcase of insertion details. Bases marked in red and blue indicate forward and reverse dsODN insertions, respectively; dark-red bases indicate recurring alignment; bases and “–” marked in black indicate mismatches between 29-bp dsODN and the nanopore read, likely due to basecall errors. **B.** and **C.** Effects of DSgrep-seq lengths on retrival rate (B) and FDR (C) of reads with dsODN insertions. Editing without dsODN serves as a control to determine the FDR. Data are represented as mean ± SEM (*n* = 24−26 independent experiments). **D.** and **E.** Effects of step values on retrieval rate (D) and FDR (E) of reads with dsODN insertions. **F.** Comparison of dsODN retrieval rates analyzed by three methods. **G.** Correlation of dsODN retrieval rates analyzed by CRISPResso2, GrepNGS, and GREPore-seq. The data in (B–F) were statistically analyzed by two-way ANOVA (adjusted *P* values were indicated). “ns” means no significance (*P* > 0.05). DNA double-strand break; WT, wild-type; GrepNGS, NGS data analyzed by both CRISPResso2 and GREPore-seq.

Having demonstrated the potential of GREPore-seq in analyzing WT long amplicon reads, we attempted to explore its applications in real-world situations. First, we used GREPore-seq to analyze insertions of a dsODN of 29 bp in length, which was benchmarked by Illumina amplicon sequencing and CRISPResso2 analysis. We designed a study to insert dsODNs at the DSBs of the *EEF2* locus via NHEJ after RNP nucleofection of human T cells [31] (Figure 5A, top). After data demultiplexing, we generated multiple lines of DSgrep-seqs using both the forward and reverse complemented short inserts. Next, we tested nine groups of DSgrep-seqs utilizing stretches of *k*-mers of 11, 13, or 15 nt and a step of 1, 3, or 5 nt. We found that the dsODN retrieval rates slightly but significantly decreased with increasing DSgrep-seq length (*P* < 0.0001, two-way ANOVA; Figure 5B). We also determined FDR using WT reads or RNP-edited samples without dsODN insertion. As expected, FDR significantly decreased with increasing DSgrep-seq length (*P* ≤ 0.0001, two-way ANOVA). The FDR was less than 0.1% for 13-nt and 15-nt DSgrep-seq data (Figure 5C). Since it had a higher dsODN retrieval rate and lower FDR, we used strings of 13 nt for the subsequent analysis. Next, we determined the optimal step values and found that 13-nt DSgrep-seq with a 1-nt step had a higher retrieval rate and lower FDR (Figure 5D and E). Therefore, DSgrep-seq of 13 nt with a step of 1 nt was used for dsODN data extraction in subsequent GREPore-seq analysis. We visualized WT and dsODN amplicon reads and observed dsODN insertions (tens of bases). We also observed imperfect and perfect dsODN insertions of multiple copies in both orientations, likely due to sequencing errors (Figure 5A, bottom).

We generated one NGS dataset and one nanopore dataset using identical edited gDNA samples with a 29-bp dsODN insertion to compare the two analytical approaches. The NGS data were analyzed by both CRISPResso2 and GREPore-seq (termed GrepNGS for clarity). In addition, we used GREPore-seq to analyze the long-range PCR amplicon nanopore data. The conventional CRISPResso2 analysis resulted in a significantly lower dsODN retrieval rate than GrepNGS (1.5-fold) and GREPore-seq (1.4-fold), likely because CRISPResso2 cannot identify insertions of shortened dsODN. Of note, the GrepNGS data were indistinguishable from the results analyzed by GREPore-seq, validating its usefulness (Figure 5F). Additionally, we observed perfect correlations between the data analyzed by CRISPResso2, GrepNGS, and GREPore-seq (*R*^2^ > 0.96; Figure 5G).

### GREPore-seq effectively detects HDR-mediated large insertions

**Figure 6:**
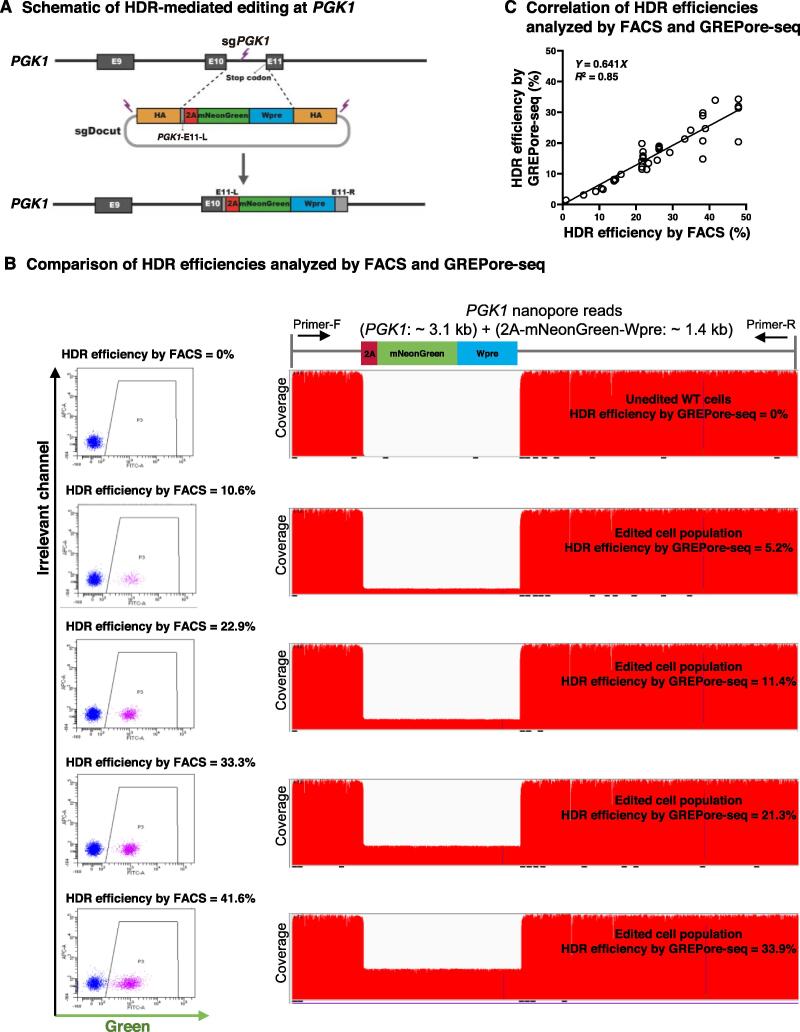
**GREPore-seq reveals the full spectrum of HDR-mediated large insertions** **A.** A schematic overview of HDR-mediated editing at *PGK1* with CRISPR/Cas9 and a double-cut donor plasmid. After precise insertion of the 2A-mNeonGreen-Wpre cassette, the cells will fluoresce in green. **B.** Tandem analysis of HDR efficiencies by FACS and GREPore-seq. Representative FACS plots and IGV presentations of PCR amplicons before and after mNeonGreen knock-in are shown. APC was an irrelevant channel showing the background signal. The proportion of mNeonGreen-positive cells indicates HDR efficiency. **C.** The linear correlation of HDR-mediated knock-ins analyzed by FACS and GREPore-seq. HA, homologous arm; 2A, 2A self-cleaving peptide; Wpre, woodchuck hepatitis virus posttranscriptional regulatory element; E9/10/11, exon 9/10/11; E11-L/R, exon 11 left/right; FACS, fluorescence-activated cell sorting.

We then assessed the utility of GREPore-seq in detecting large insertions. We designed a double-cut promoterless HDR plasmid donor and a single guide RNA (sgRNA) targeting the *PGK1* stop codon. After precise HDR-mediated editing, iPSCs fluoresce in green (Figure 6A) [32]. We chose the *PGK1* gene for editing in a male (XY) iPSC line because *PGK1* is a housekeeping gene located on chromosome X. Therefore, all mNeonGreen^+^ cells will have one HDR allele and no WT allele, allowing for independent verification by fluorescence-activated cell sorting (FACS) analysis. To facilitate correlation analysis, we mixed WT and high-level edited cells at different ratios to create populations with mNeonGreen^+^ cells ranging from 0% to 50%. For GREPore-seq analysis, we PCR-amplified an ∼ 3.5-kb WT allele or an ∼ 4.5-kb HDR allele, followed by pooled nanopore sequencing. HDR-mediated editing of *PGK1* led to the insertion of an ∼ 1.4-kb donor and deletion of the ∼ 400-bp intron (sequence details are shown in Table S2). HDR-mediated editing was visualized by mapping the expected HDR allele reference and quantitated by counting large deletions in the compact idiosyncratic gapped alignment report (CIGAR) strings [33]. As a control, the unedited WT cells showed 0% mNeonGreen knock-in by FACS analysis or GREPore-seq (Figure 6B). FACS analysis indicated that HDR efficiencies were 10.6%, 22.9%, 33.3%, and 41.6%, while GREPore-seq displayed efficiencies of roughly 5.2%, 11.4%, 21.3%, and 33.9%, respectively. The HDR efficiencies analyzed by GREPore-seq were slightly lower than those assessed by FACS, attributed to less effective amplification of longer amplicons (3.5 kb *vs.* 4.5 kb for WT allele *vs.* HDR allele). Nevertheless, we observed an excellent linear correlation between the HDR-mediated knock-in efficiencies analyzed by FACS and GREPore-seq (*R*^2^ = 0.85; Figure 6C).

### GREPore-seq accurately detects NHEJ-mediated plasmid BB insertions

**Figure 7:**
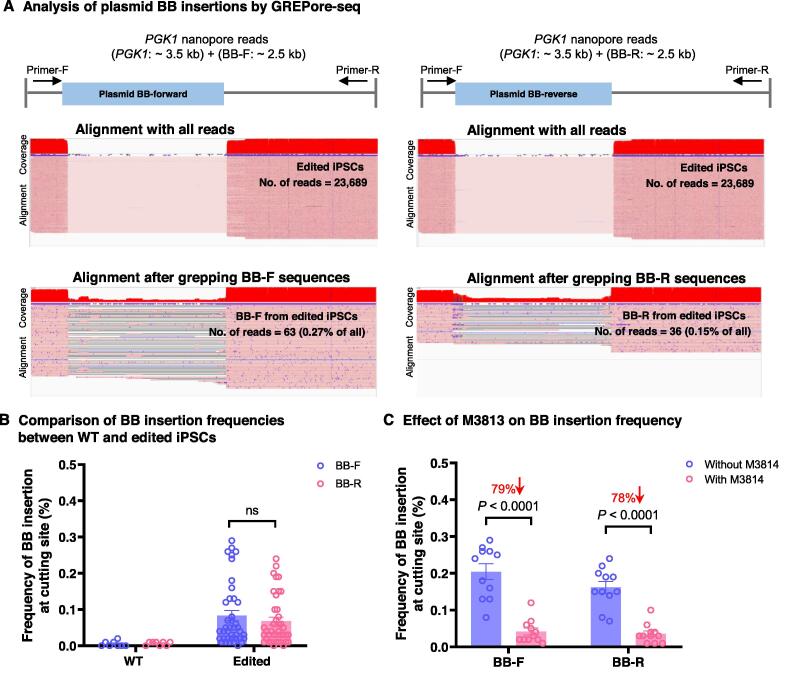
**GREPore-seq discovers NHEJ-mediated plasmid BB insertions** **A.** Trace amount of plasmid BB insertion at the CRISPR cutting site after HDR-mediated editing in iPSCs. Top: schematic of forward or reverse insertion of the BB sequence. Middle: almost invisible number of reads with BB insertion. Bottom: visualization of the details after enriching reads with fragmented or full-length BB insertion. **B.** Frequencies of BB-F and BB-R in WT and edited iPSCs. Unedited WT cells serve as a negative control. Data are represented as mean ± SEM (*n* = 8 independent experiments in WT iPSCs and *n* = 40 independent experiments in edited iPSCs). **C.** Effect of M3814 on plasmid BB insertion frequency. NHEJ inhibition with M3814 considerably reduced plasmid BB insertion. Data are represented as mean ± SEM (*n* = 11 independent experiments). The data in (B) and (C) were analyzed by two-way ANOVA (adjusted *P* values were indicated). “ns” means no significance (*P* > 0.05). BB-F, forward plasmid backbone insertion; BB-R, reverse plasmid backbone insertion; NHEJ, non-homologous end-joining; iPSC, induced pluripotent stem cell.

The double-cut donor might induce insertion of the plasmid BB via the NHEJ pathway [34]. Therefore, we further assessed the extent to which the plasmid BB ( ∼ 2.5 kb) was inserted in the above-edited iPSCs using a double-cut HDR donor (sequence details are shown in Table S2). For this purpose, we generated grep-seqs of 15 nt with a step of 100 nt using the forward and reverse complemented plasmid BB sequences. After GREPore-seq processing, we observed insertions of plasmid BB at the cutting site in both forward and reverse orientations. We identified 63 reads of forward plasmid BB insertions (BB-F) and 36 reads of reverse plasmid BB insertions (BB-R) in a total of 23,689 reads (0.27% and 0.15%, respectively; Figure 7A). Of note, 10%−20% of insertions were of the full-length BB, with the rest being fragmented sequences. WT amplicons showed virtually 0% plasmid BB insertions (Figure 7B). Furthermore, consistent with the notion of NHEJ-dependent plasmid BB insertion, inhibition of the NHEJ pathway with M3814 significantly decreased its insertion by ∼ 80% to a level of 0.04% (Figure 7C). As such, we are the first to illustrate a full spectrum of plasmid BB insertions after HDR-mediated editing using our GREPore-seq workflow.

### Features and implementation of GREPore-seq

**Figure 8:**
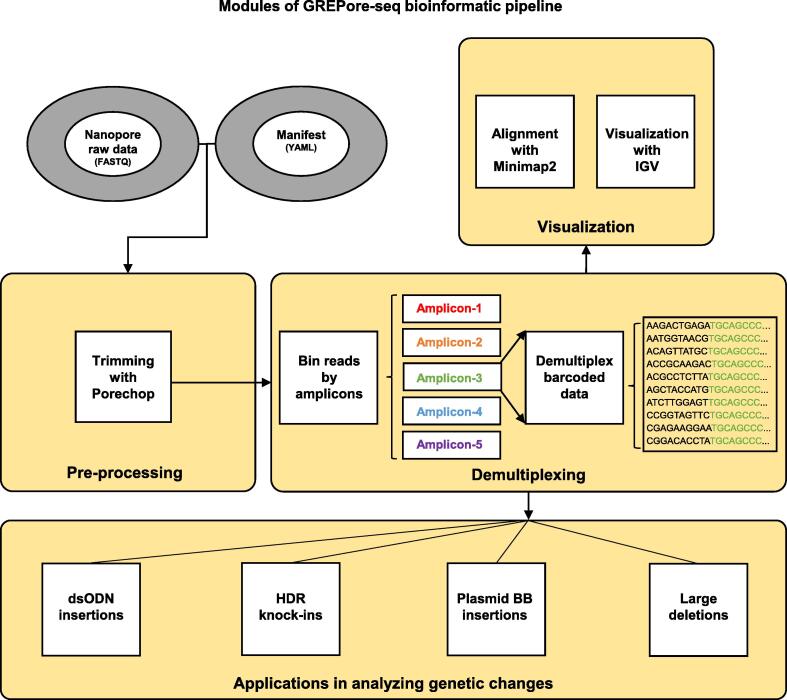
**Schematic of the GREPore-seq workflow** There are four modules in the GREPore-seq bioinformatic pipeline, including pre-processing, demultiplexing, visualization, and an applications-in-analyzing-genetic-changes module.

The GREPore-seq pipeline consists of a pre-processing module, a demultiplexing module, a visualization module, and an applications-in-analyzing-genetic-changes module. The pre-processing module takes raw reads from a pooled multisite nanopore sequencing run as input to trim adaptors. The second module demultiplexes reads into amplicon-specific and barcode-specific data. Then, the demultiplexed reads align with references using Minimap2, followed by visualization with IGV. Finally, the analysis module uses GREPore-seq to quantify dsODN insertions, HDR knock-ins, plasmid BB insertions, and large deletions (Figure 8). Our recently study has detailed the assessment of large deletions after CRISPR/Cas9 editing [31].

The GREPore-seq bioinformatic pipeline can be implemented using a desktop or laptop computer with 16- or 64-GB RAM. In typical examples, it took 0.5 h to process raw data of ∼ 2 GB with 60 amplicons and 3 h to finish analyzing 10-GB data with 120 amplicons on a 64-GB RAM desktop computer using a single processor.

## Discussion

An increasing amount of studies have used nanopore sequencing to interrogate basic and translational research questions that NGS is otherwise impossible. However, the high error rate hampered its broad applications. In addition, it is ∼ 5 times more expansive than Illumina sequencing in terms of data quantity. To make 3GS accessible to more laboratories, one needs to pool more samples and create a novel bioinformatic pipeline aware of the unique features of all ONT data. Here, we have advanced this field by solving multiple problems: (1) efficiently generating long amplicons, (2) retrieving a pool of full-length amplicons with high recovery and low FDR, and (3) choosing indel-correcting barcodes for effective data binning. Along the way, we developed the GREPore-seq pipeline to facilitate data analysis. We also demonstrated identifying indels of over one kilobase. Finally, we discovered the integration of full-length and fragmented plasmid BB sequences at the editing site for the first time.

In the beginning, we optimized the barcoded long-range PCR protocol. We found that PrimeSTAR can amplify amplicons of diverse sizes and is the most robust in specificity and productivity among the three commercial kits we assessed, which may attribute to its improved DNA polymerase in PrimeSTAR. In this study, PrimeSTAR GXL DNA polymerase generated 4–8-kb amplicons effectively using the recommended protocol [31]. After further optimization, we speculate that this system can also amplify 10–20-kb PCR products. In addition, we found that tagging a barcode of 10–14 nt did not significantly affect PCR amplification, allowing for pooled nanopore sequencing.

A barcode of 20–24 nt was added to multiplex samples for nanopore sequencing during library construction. However, this is labor-intensive and costly. In contrast, barcode tagging by PCR is more affordable and easier to scale up. Considering the high-level indel mutation errors of the nanopore data, we decided to use barcodes that tolerate 2-nt mismatches and indels. DNA barcodes were added to 5′-end of the forward primers to obtain barcoded amplicons. We used indel-correcting DNA barcodes [23] to correct substitution, insertion, and deletion errors of up to 2 nt. This study pooled up to ∼ 120 PCR products for nanopore sequencing, and GREPore-seq can process the data efficiently with negligible FDR. We recommend using 10-nt, 12-nt, and 14-nt barcodes, for which up to 60, 350, and 2300 indel-correcting barcodes, respectively, are available [23]. Therefore, indel-correcting DNA barcodes are a convenient tool for multiplex nanopore sequencing.

In nanopore sequencing, ∼ 5% of reads are longer, and ∼ 40% are significantly shorter than the expected amplicon size, likely due to aborted sequencing or base-calling errors. Thus, to obtain a reliable result, removing questionable data, in particular, truncated reads, is needed. By capturing signature sequences at both ends of the references, we showed that the proportion of longer and shorter reads dropped to ∼ 2% and 10%, respectively. As a result, this nanopore data retrieval scheme decreases the FDR by ∼ 4 folds.

Nanopore reads usually consist of a stretch of ∼ 20 correctly called bases, gapped with one or more mismatches and/or indels. Of note, these errors are primarily random. With these features in mind, we developed a new algorithm to bin the amplicon-specific reads: we generated multiple lines of overlapping (5–10 nt) reference amplicon sequences of 15 nt with a progress step of 5 nt, located 20–90 nt away from the amplicon ends, excluding the primer sequence of ∼ 20 nt. The use of ∼ 10 overlapped grep sequences overcomes the nanopore’s intrinsic error of short indels. Using this approach, we showed a data retrieval rate of up to 70% and an FDR of less than 0.1%. To ensure accurate locations of signature sequences, we used Porechop to remove adaptor sequences in the pre-processing module. We expect this indel-aware nanopore reads retrieval regimen to find wide applications in analyzing 3GS data.

This work developed a GREPore-seq data analysis pipeline dedicated to demultiplexing and visualizing nanopore data. It was written with Python and can be readily implemented on personal computers. This pipeline can assess different types of large insertions and large deletions. Previously, large insertions or knock-ins introduced by HDR were analyzed at the RNA or protein levels by Northern blotting, Western blotting [35], or flow cytometry [34]. However, the accuracy of full-length insertions has not been evaluated. Using GREPore-seq, we demonstrated that HDR-mediated editing leads to precise integration of the insert that can be visualized at a single-nucleotide level. Of interest, we also captured a trace amount of plasmid BB insertions at the CRISPR target site, highlighting the power of 3GS in understanding the full spectrum of editing outcomes.

Template plasmid integration events have been detected on the genome-edited calves by whole-genome sequencing previously [36]. We identified low yet detectable levels of full-length plasmid BB insertion in edited cells by long-range PCR and nanopore sequencing. This has potential applications in identifying correctly edited iPSC clones for clinical therapies. Undoubtedly, insertion of other HDR donor templates, such as ssODN and adeno-associated virus (AAV), can also be assessed by GREPore-seq. We also reported that inhibition of the NHEJ pathway leads to considerably lower unintended insertion of the vector BB. An accompanying paper published in *Genome Biology* showed that GREPore-seq could evaluate loss-of-heterozygosity by examining single-nucleotide polymorphisms (SNPs) and large deletions after CRISPR/Cas9 editing [31].

We also showed that GREPore-seq could quantify insertions of short 29-bp dsODNs. In applications that are considerably different from a short insertion, one may readily adjust the DSgrep-seq parameters to achieve optimal data retrieval and minimal FDR, as we showed in Figure 5. However, GREPore-seq was not recommended to detect very short (*i.e.*, 1–6 bp) insertions or mutations. In this case, NGS technology combined with data analysis software such as CRISPResso2 is still valuable [4].

Due to the popularity and cost-effectiveness of nanopore sequencing, we designed a GREPore-seq workflow to process ONT sequencing data. We have also optimized a multiplex sequencing protocol to reduce the cost for each sample further. Undoubtedly, GREPore-seq can also analyze PacBio sequencing data with similarly high error rates. Of note, the latest PacBio Sequel II system can perform circular consensus sequencing and generate accurate (> 99%) high-fidelity (HiFi) reads [37], which will simplify the analysis procedure. In comparison, the average accuracy of ONT 1D^2^ reads is up to 95% (R9.5 nanopore) [38].

Our work has limitations. This study is based on long-range PCR followed by nanopore sequencing, which may have potential PCR amplification bias. The HDR efficiencies detected by GREPore-seq were slightly lower than the results benchmarked by FACS analysis. Similarly, large deletions might be overestimated when amplifying the target site in a pool of edited alleles with different sizes. However, by increasing the length of the amplicon and thereby decreasing the ratio of WT and edited allele length, this bias can be partially mitigated.

## Conclusion

In summary, we have developed an experimental procedure and bioinformatic pipeline to analyze and visualize genetic changes after CRISPR/Cas9-mediated genome editing, including on-target knock-in or deleterious insertion of template sequences and large deletions. This approach allows for pooling large quantities of PCR amplicons, thus being suitable for large-scale and low-cost data analysis. The new algorithm also increases the recovery of full-length amplicons with a low FDR.

## Materials and methods

### Cell culture

#### K562 cells

K562 cells (American Type Culture Collection) were cultured in RPMI-1640 medium (Catalog No. 22400089, Gibco, Grand Island, NY) supplemented with 10% fetal bovine serum (FBS; Catalog No. 10099141, Gibco, Australia). Cells were passaged twice a week.

#### Human primary T cells

CD3^+^ T cells were isolated from peripheral blood mononuclear cells (PBMCs) and expanded as previously described [31]. T cells were grown in nontissue culture-treated 6-well plates, with medium changed every two days.

#### Human HSPCs

CD34^+^ HSPCs were collected from cord blood and expanded as previously described [31]. Over 90% purity of CD34^+^ cells were detected in the enriched HSPCs.

#### Human iPSCs

iPSCs were produced by reprogramming PBMCs and cultured as previously reported [31], [39], [40]. To increase cell survival, the ROCK inhibitor Y-27632 (10 M; LC Laboratories) was added during the first 24 h following passaging with Accutase (Catalog No. AT-104, Innovative Cell Technologies).

All of the cells mentioned above were cultured in a humidified atmosphere with 5% CO_2_ at 37 °C.

### *gRNA design*

We used CHOPCHOP [41], [42] to design appropriate gRNAs targeting human *AAVS1*, *B2M*, *BCL11A-2/BCL11A-4*, *EEF2*, *TRBC*, and *PGK1*. sg*BCL11A-1* and sg*BCL11A-3* were previously reported to target the *BCL11A* GATA motif [43]. sg*TRAC* was previously reported to target the endogenous T cell receptor (TCR) α chain [44]. Table S3 lists the gRNAsused in this study.

### Plasmid construction

NEBuilder HiFi DNA Assembly Master Mix (Catalog No. E2621L, New England Biolabs) was used to assemble all Cas9, sgRNA, and donor plasmids from specific liner DNA products. Trans5α Chemically Competent Cells (Catalog No. CD201-02, TransGen, Beijing, China) were subsequently transformed with the produced products and ampicillin-plated on LB agar plates. Finally, to select the correct clones, we chose multiple colonies for Sanger sequencing (Tsingke Biotechnology).

### RNP formation

crRNAs and tracrRNA with chemical modifications were synthesized by Synthego or Integrated DNA Technologies (IDT) and demonstrated comparable efficacies. Alt-R SpCas9 Nuclease V3 protein (Catalog No. 1081059, IDT) contained a nuclear localization sequence. The gRNA complex was annealed according to the instructions. Cas9 RNPs were freshly produced by combining Cas9 protein (60 pmol) with gRNA (150 pmol) at a molar ratio of 1:2.5 at room temperature for 10–20 min prior to electroporation.

### Gene editing

For transfection of K562 cells with editing components, program T-016 of 2b-nucleofector and 70 μl Amaxa Cell Line Nucleofector Kit V (Catalog No. VVCA-1003, Lonza) were used. In previous studies, we outlined the methodologies for electroporating Human T cells, HSPCs, and iPSCs [31], [32], [45].

### Flow cytometry

The HDR efficiencies of mNeonGreen-positive cells were evaluated using flow cytometry, as described previously [32], [45], [46]. After 3 days of nucleofection, cells were detected using a BD FACS Canto II flow cytometer. We categorized the fluorescence-positive cell population as HDR knock-in cells that had the promoterless mNeonGreen reporter inserted into the *PGK1* target site. Cells transfected without gRNA or the plasmid donor served as negative controls, showing 0% mNeonGreen positivity (Figure 6B).

### Long-range PCR and barcoding

Cells were harvested for gDNA extraction 3 days after transfiguring gene-editing components using the QIAamp DNA Mini Kit (Catalog No. 51306, Qiagen, Hilden, Germany) following the recommended instructions. To evaluate the performance of KAPA HiFi DNA Polymerase (Catalog No. KK2602, Kapa Biosystems, Cape Town, South Africa), NileHiFi Long Amplicon PCR Kit (Catalog No. PC002, GeneCopoeia, Rockville, MD), and PrimeSTAR GXL DNA polymerase, each of the three long-range PCR enzymes was used to amplify the same wild-type gDNA sample. All experiments were conducted using the cycling conditions recommended by the manufacturers.

Subsequently, we amplified all target sequences by PrimeSTAR GXL DNA polymerase. The 10-μl PrimeSTAR PCR system contained 100 ng of extracted gDNA, 2× premix, and 0.3 μl primer (10 μM). For 30 cycles of PCR, the temperature was set to 98 °C for 10 s, 60 °C for 15 s, and 68 °C for 1 min per kb. For relatively low-specificity primers, we took 5 cycles of touch-town PCR (65 °C, −1 °C/cycles) and 25 cycles of the aforementioned standard procedure. The 8–14-nt indel-correcting DNA barcodes [23] were added at 5′-end of the forward primers. The barcode primers used in this study are listed in Table S4. And we generally mixed multiple PCR products with different barcodes for nanopore sequencing.

### Nanopore sequencing

The 1D library preparation used input 8 μg of long-range PCR amplicons per sample by ligation SQK-LSK109 Kit (ONT, UK), and then added the sequencing adaptor, motor protein, and tether protein after end repair and A-tailing. PromethION (ONT, UK) at Novogene (Tianjin, China) was used to sequence the library. Albacore (v2.3.1; ONT) was used to transform raw fast5 data into a FASTQ file format, which contains the sequence information of the reads and the corresponding quality information.

### Data processing using the GREPore-seq workflow

The commands used to process the data are detailed below. All the following steps have been integrated into the GREPore-seq Python script.

#### Pre-processing: trimming raw data with Porechop

Raw fastq nanopore data were processed by Guppy, followed by adaptor trimming using Porechop (v0.2.4; https://github.com/rrwick/Porechop) with the command “--adapter_threshold 85 --extra_end_trim 0”.

#### Demultiplexing barcoded data into target-specific data

We grepped data using signature sequences of ∼ 70 bp at both ends of the references to remove partially sequenced reads, omitting the primer sequences. Stretches of *k*-mers with 15 nt were generated from signature sequences with a step size of 5 nt. In other words, each 15 nt *k*-mer overlaps 10 nt with the next one. These sets of 15-mers are termed Grepseq-left or Grepseq-right. To demultiplex barcoded amplicons, we similarly generated BCseqs, but with barcode and primer sequences as the signature sequences. Approximately 5–10 9-mers with a step size of 1 nt were generated.

All reads with more than 2 matches (*n* ≥ 2) to the *k*-mers in Grepseq-left, Grepseq-right, and BCseqs were binned together and written into new fastq files, named by their sample ID.

#### Visualizing the demultiplexed data

The nanopore reads were first aligned with reference amplicon sequences. Mapping was performed with Minimap2 (v2.17; GitHub - lh3/minimap2) [26] using command “minimap2 -ax map-ont Reference.mmi amplicon.fastq.gz > amplicon.sam”; and sorted with SAMtools (v1.10; GitHub - samtools/samtools) [33] using the following commands: (1) SAMtools view “-bS amplicon.sam > amplicon.bam”; (2) SAMtools sort “-O bam -o amplicon.sorted.bam -T temp amplicon.bam”; and (3) SAMtools index “amplicon.sorted.bam”. After processing, the sorted bam and index files for each demultiplexed amplicon-specific dataset were then used for visualization with IGV or used for subsequent analysis.

### Analysis of genetic changes

#### dsODN insertions

To identify 29-bp dsODN insertions at the Cas9-gRNA cleaving sites with the GREPore-seq workflow, we used stretches of *k*-mers termed DSgrep-seqs. Similar to Grepseqs and BC-primer-seqs, DSgrep-seqs were generated from the 29-bp dsODN in both orientations using a sliding window size of 13 nt and a step size of 5 nt. If more than one (*n* ≥ 2) 13-mer in DSgrep-seqs matched the reads in the amplicon-specific fastq file, the data were binned to a new file named “ID–DSgrep”. Finally, we calculated the dsODN insertion rate, defined as the ratio of reads carrying DSgrep-seq sequences to the total number of amplicon-specific reads. To benchmark the DSgrep-seq results, the same edited samples with dsODN insertions were subjected to Illumina sequencing. CRISPResso2 (v2.1.1; GitHub - pinellolab/CRISPResso2) was used to compute the dsODN insertion rates.

#### HDR-mediated editing

To assess the HDR-mediated gene knock-in efficiency, we calculated the soft-clipped reads of the WT sequence in the CIGAR strings supplied in SAMtools format. Match, alignment gap, deletion, and insertion are some of the operations that make up a CIGAR string. Initial mapping algorithms can deal with indels shorter than 50 bp and allow gaps longer than several hundred base pairs by Minimap2. Reads that can’t be fully mapped, as well as their alignments, frequently have a mix of matched and mismatched sections. In the CIGAR strings given in the SAMtools format, the latter is referred to as soft-clipped [26], [33], [47]. We counted CIGAR-S for every amplicon-specific alignment mapped with the HDR allele reference using a custom Python script to quantify knock-in with over a thousand bases. If the CIGAR-S was greater than 1000, it was binned to the output, which was then divided by the total number of alignments to compute artificial “deletion” efficiency. Since the expected HDR-edited sequence was used as a reference for alignment, a WT allele will artificially display a large deletion. Therefore, the HDR efficiency was calculated as 100% substracted by the artificial “deletion” efficiency.

#### Plasmid BB insertions

To identify plasmid BB insertions in edited cells, we grepped the reads with stretches of BB *k*-mers generated using a window size of 15 nt and a step size of 100 nt. The plasmid BB insertion rates were defined as the ratio of reads with BB insertions to the total number of amplicon-specific reads.

### Illumina sequencing and analysis of editing efficiency

To avoid artifacts caused by the donor plasmid, primary PCR was performed with primers that targeted genomic regions aligned outside the homology arms. The primary PCR products were diluted 100× and utilized as templates for subsequent PCR, resulting in 200–240-bp amplicons for Illumina paired-end 150 bp sequencing. The secondary PCR was performed with KAPA HiFi polymerase under the following cycling conditions: 98 °C for 1 min, followed by 98 °C for 5 s, 64 °C for 10 s, and 72 °C for 15 s for 20 cycles. As previously stated, barcoded primers were employed [32], [48]. For stringent comparison, the primary long PCR products were sequenced by ONT, and the secondary short amplicons were analyzed with an Illumina sequencer. Table S5 lists the PCR primers used in this work. The paired-end fastq data were merged with FLASH (v1.2.11) [49] or data processing, followed by demultiplexing with Barcode-splitter (v0.18.6). The docker version of CRISPResso2 [13], [14] was used to examine indel frequencies, HDR efficiencies, and dsODN insertion rates.

### Statistics and reproducibility

We used two-way ANOVA to evaluate paired/matched or unmatched data. GraphPad Prism v8.0.1 (GraphPad Software, La Jolla, CA) was used to calculate the *P* values. Adjusted *P* values were indicated. The letter “ns” stands for “no significance” (*P* > 0.05). All of the data presented came from at least three independent experiments.

## Code availability

The code of GREPore-seq is publicly available at GitHub (https://github.com/lisiang/GREPore-seq) and BioCode (https://ngdc.cncb.ac.cn/biocode/tools/BT007293).

## Data availability

Nanopore and Illumina sequencing data in this study have been deposited in the Genome Sequence Archive for Human [50] at the National Genomics Data Center, Beijing Institute of Genomics, Chinese Academy of Sciences / China National Center for Bioinformation (GSA-Human: HRA001801 for Nanopore sequencing data and HRA001802 for Illumina sequencing data), and are publicly accessible at https://ngdc.cncb.ac.cn/gsa-human/.

## Competing interests

The authors have declare no competing interests.

## CRediT authorship contribution statement

**Zi-Jun Quan:** Conceptualization, Methodology, Formal analysis, Investigation, Writing – original draft. **Si-Ang Li:** Methodology, Software, Data curation, Writing – original draft. **Zhi-Xue Yang:** Validation, Resources. **Juan-Juan Zhao:** Validation. **Guo-Hua Li:** Resources. **Feng Zhang:** Resources. **Wei Wen:** Conceptualization, Methodology, Formal analysis, Investigation, Writing – review & editing, Supervision. **Tao Cheng:** Supervision, Funding acquisition. **Xiao-Bing Zhang:** Conceptualization, Writing – review & editing, Supervision, Project administration, Funding acquisition. All authors have read and approved the final manuscript.

## Supplementary material

Supplementary Table S1 **Reference position in GRCh38/hg38 for nanopore sequencing data alignment**

Supplementary Table S2 **Alignment reference sequences of *PGK1* after insertions**

Supplementary Table S3 **Guide RNA sequences**

Supplementary Table S4 **Forward primers with/without barcodes and the reverse primer for nanopore sequencing data**

Supplementary Table S5 **Forward primers with barcodes and the reverse primer for Illumina paired-end 150 bp sequencing data**
