# Supplementary material for: GREPore-seq: A Robust Workflow to Detect Changes After Gene Editing Through Long-range PCR and Nanopore Sequencing
Source: Genomics Proteomics Bioinformatics. 2022 Jun 23;21(6):1221–36. doi: 10.1016/j.gpb.2022.06.002 (PMC11082256; doi:10.1016/j.gpb.2022.06.002)
Supplement: Supplementary Table S1 — Reference position in GRCh38/hg38 for nanopore sequencing data alignment [file mmc1.docx]

**Table S1 Reference position in GRCh38/hg38 for nanopore sequencing data alignment**

| **Site name** | **Position in** **GRCh38/hg38** |
| --- | --- |
| *AAVS1* | Chr19: 55,113,029 - 55,116,956 |
| *BCL11A-3* | Chr2: 60,493,622 - 60,497,484 |
| *BCL11A-4* | Chr2: 60,517,886 - 60,523,198 |
| *EEF2* | Chr19: 3,975,169 - 3,980,455 |
| *PGK1* | ChrX: 78,124,787 - 78,128,338 |
