# Supplementary material for: GREPore-seq: A Robust Workflow to Detect Changes After Gene Editing Through Long-range PCR and Nanopore Sequencing
Source: Genomics Proteomics Bioinformatics. 2022 Jun 23;21(6):1221–36. doi: 10.1016/j.gpb.2022.06.002 (PMC11082256; doi:10.1016/j.gpb.2022.06.002)
Supplement: Supplementary Table S2 — Alignment reference sequences of PGK1 after insertions [file mmc2.docx]

**Table S2 Alignment reference sequences of *PGK1* after insertions**

*Note*: red sequences represent insertions.

| Types | Sequences |
| --- | --- |
| Forward mNeonGreen insertion | TGCAGCCCTGAGTTCTGGTCTTGGTGGAGGTGGTGTTTAAGTAGCTTTTCTTGATAGCTCATCTTCTCTTTCACCTCTACCCCTCAGGGCTTGGACTGTGGTCCTGAAAGCAGCAAGAAGTATGCTGAGGCTGTCACTCGGGCTAAGCAGATTGTGTGGAATGGTCCTGTGGGGGTATTTGAATGGGAAGCTTTTGCCCGGGGAACCAAAGCTCTCATGGATGAGGTGGTGAAAGCCACTTCTAGGGGCTGCATCACCATCATAGGTAAGCGGTCCTATACAAAGCTAATACCCATATAAGCTGGCAGAATTCTGATCAGAGGAAGGTGGAATGGAGAACTTCTTCTATGTCTCTTTATTCTGGGTAAATGTTAAGAGGTAAACAGGTAGGTAATTTACAGAGGAGCCTCTTGGTAAGATAGAGTTGGGGGTTTATCAGCTACCTTTTGGGTTGGGGAGCACACTGCCTTACAGTTTTGGTGCCAATCCCTTTTTTTTCTTTTCTCTCTTTTCCCTTTTTACCTGGCTTTCATTCAACAGGTGGTGGAGACACTGCCACTTGCTGTGCCAAATGGAACACGGAGGATAAAGTCAGCCATGTGAGCACTGGGGGTGGTGCCAGTTTGGAGCTCCTGGAAGGTAAAGTCCTTCCTGGGGTGGATGCTCTCAGCAATATTGCTAGCCAGTGTACTAATTATGCTCTCTTGAAATTGGCTGGAGATGTTGAGAGCAACCCAGGTCCCATGGTGAGCAAGGGCGAGGAGGATAACATGGCCTCTCTCCCAGCGACACATGAGTTACACATCTTTGGCTCCATCAACGGTGTGGACTTTGACATGGTGGGTCAGGGCACCGGCAATCCAAATGATGGTTATGAGGAGTTAAACCTGAAGTCCACCAAGGGTGACCTCCAGTTCTCCCCCTGGATTCTGGTCCCTCATATCGGGTATGGCTTCCATCAGTACCTGCCCTACCCTGACGGGATGTCGCCTTTCCAGGCCGCCATGGTAGATGGCTCCGGATACCAAGTCCATCGCACAATGCAGTTTGAAGATGGTGCCTCCCTTACTGTTAACTACCGCTACACCTACGAGGGAAGCCACATCAAAGGAGAGGCCCAGGTGAAGGGGACTGGTTTCCCTGCTGACGGTCCTGTGATGACCAACTCGCTGACCGCTGCGGACTGGTGCAGGTCGAAGAAGACTTACCCCAACGACAAAACCATCATCAGTACCTTTAAGTGGAGTTACACCACTGGAAATGGCAAGCGCTACCGGAGCACTGCGCGGACCACCTACACCTTTGCCAAGCCAATGGCGGCTAACTATCTGAAGAACCAGCCGATGTACGTGTTCCGTAAGACGGAGCTCAAGCACTCCAAGACCGAGCTCAACTTCAAGGAGTGGCAAAAGGCCTTTACCGATGTGATGGGCATGGACGAGCTGTACAAGTAAGTTTAAACGTCGACAATCAACCTCTGGATTACAAAATTTGTGAAAGATTGACTGGTATTCTTAACTATGTTGCTCCTTTTACGCTATGTGGATACGCTGCTTTAATGCCTTTGTATCATGCTATTGCTTCCCGTATGGCTTTCATTTTCTCCTCCTTGTATAAATCCTGGTTGCTGTCTCTTTATGAGGAGTTGTGGCCCGTTGTCAGGCAACGTGGCGTGGTGTGCACTGTGTTTGCTGACGCAACCCCCACTGGTTGGGGCATTGCCACCACCTGTCAGCTCCTTTCCGGGACTTTCGCTTTCCCCCTCCCTATTGCCACGGCGGAACTCATCGCCGCCTGCCTTGCCCGCTGCTGGACAGGGGCTCGGCTGTTGGGCACTGACAATTCCGTGGTGTTGTCGGGGAAGCTGACGTCCTTTCCATGGCTGCTCGCCTGTGTTGCCACCTGGATTCTGCGCGGGACGTCCTTCTGCTACGTCCCTTCGGCCCTCAATCCAGCGGACCTTCCTTCCCGCGGCCTGCTGCCGGCTCTGCGGCCTCTTCCGCGTCTCGCCTTCGCCCTCAGACGAGTCGGATCTCCCTTTGGGCGGATCCTAGTACTTTCCTGCCTTTTAGTTCCTGTGCACAGCCCCTAAGTCAACTTAGCATTTTCTGCATCTCCACTTGGCATTAGCTAAAACCTTCCATGTCAAGATTCAGCTAGTGGCCAAGAGATGCAGTGCCAGGAACCCTTAAACAGTTGCACAGCATCTCAGCTCATCTTCACTGCACCCTGGATTTGCATACATTCTTCAAGATCCCATTTGAATTTTTTAGTGACTAAACCATTGTGCATTCTAGAGTGCATATATTTATATTTTGCCTGTTAAAAAGAAAGTGAGCAGTGTTAGCTTAGTTCTCTTTTGATGTAGGTTATTATGATTAGCTTTGTCACTGTTTCACTACTCAGCATGGAAACAAGATGAAATTCCATTTGTAGGTAGTGAGACAAAATTGATGATCCATTAAGTAAACAATAAAAGTGTCCATTGAAACCGTGATTTTTTTTTTTTTCCTGTCATACTTTGTTAGGAAGGGTGAGAATAGAATCTTGAGGAACGGATCAGATGTCTATATTGCTGAATGCAAGAAGTGGGGCAGCAGCAGTGGAGAGATGGGACAATTAGATAAATGTCCATTCTTTATCAAGGGCCTACTTTATGGCAGACATTGTGCTAGTGCTTTTATTCTAACTTTTATTTTTATCAGTTACACATGATCATAATTTAAAAAGTCAAGGCTTATAACAAAAAAGCCCCAGCCCATTCCTCCCATTCAAGATTCCCACTCCCCAGAGGTGACCACTTTCAACTCTTGAGTTTTTCAGGTATATACCTCCATGTTTCTAAGTAATATGCTTATATTGTTCACTTCTTTTTTTTTTATTTTTTAAAGAAATCTATTTCATACCATGGAGGAAGGCTCTGTTCCACATATATTTCCACTTCTTCATTCTCTCGGTATAGTTTTGTCACAATTATAGATTAGATCAAAAGTCTACATAACTAATACAGCTGAGCTATGTAGTATGCTATGATTAAATTTACTTATGTAACTTTTATTGTCTTTGGCATTAACAGTGTTTCAAAAAATTTTCTGTGTATACCCATCAGTGATTCATTCCCAAATCTTCTAGAAGCATAAGTGTCTCAATATATTAAAACATATTGAATAATCCTTGTTAGAGTTATCCCTGCAGGAGTCCTTAGTGCTCCTTTATCCAATTTGTACTTGATGCCCTCTAGGCAGGGTGTACAGCTAGCTGTTGCTCTGGTATTTCCTATAACCTTCTTGGGGATTTCTTTTACCTCCTGTGTTAGACTCCTGTTTTCTGGATTCCCCCTTTTCCCTCTTTCTTGGTCTACTTTTTGTAGAACACAAGACTCTACTAGCTTCCTGAGAAAGGGTGCCTGGGAGGCAAAATCTCTAAGACTTTGTAAGTCTGAAAATGTCTTTATTCGACCCTTATACTTGATTCCTAGTTTGGCTATATATAGAATTTTAGCCTGAGTATCACTTTTTGAGACTCGAAGCCACTGTTTCATTGTCACTATTGAGAATCTAAATGGCCATTCGGATTCTTTCATCATCTTTATAATTTTACCTTCCTATTTCTTTCCTGTCCTTTTAATGGAGTTTTGGGAGAGAGCAGAGGTAAACATGATTTGAAAAAGCCATGTCTGACCAGAAATTCTGTGCTGGAAAGATATGTATTTCACCTTTAGGGACAAAGAAATAAACTTTTGGACAGGACCACAGAGCTAGTAAGTAACAGCAGGGATTCAAGTCCAGGTTTGTCTGGTTCCAGTGGCTGATGCTTTTCCAATGTGCCTCCGTCCCTGACATGATGCTTCTAGGCTATAGATGCTTCTAGACTCTATCCCTGACATGATGCTTCTAGACTATTTTGTTTAACCCTGGATAGAATAGCAAAAGAAAATTTTGGTGGTTGCTCTAAACAAAACAGAAATTTGAAAGCTCAAGTTTTTTCTTCATTTGTATTTTAGTTAATACTGTACCCATATTTGTAGTTAATTTTAAATTGTACCATGTTTCTGCATACCTCTATGGGTACTCAGGAATTCTAGTCCAATTTTTGTGACTTTTTCCTACTGATTACCTTTCCTCCAACGTTTTAAAAATTATTTCAAATGGAACTGAAAGAAGCATAAACTCCCATAAACCCAGCACCTAGACTCTACAATTGCCAACATTATCTATCCAACAATCTCTGACTGTACCTTTTAAGTACTTTCTACTGGACTGTTTCCAGGATCACCCCTCATTTATTTGGGTTGTTAACCTAAAGAATGAATGGGGGAATCTCCAGTCATAAACAACTTGTCAATTAGGCAAATATTTGAGTTCCTTCTATGTGCTTAAAGACGTGATAGAGGGAATATAAGAGCATTTATGTTCTGAAGGAATTTTTAACCTAACCAAAGAAGATAGTAGATAACTTGTGTGCATATAAGCTAGAACAGTATAAGGGGCCGGGCATGGTGGCTTACGCCTGTAATCCCAGGACTTTGGGAGGCCAAGGCGGGCAGATCACCTGTCAGGAGT |
| Reverse mNeonGreen insertion | TGCAGCCCTGAGTTCTGGTCTTGGTGGAGGTGGTGTTTAAGTAGCTTTTCTTGATAGCTCATCTTCTCTTTCACCTCTACCCCTCAGGGCTTGGACTGTGGTCCTGAAAGCAGCAAGAAGTATGCTGAGGCTGTCACTCGGGCTAAGCAGATTGTGTGGAATGGTCCTGTGGGGGTATTTGAATGGGAAGCTTTTGCCCGGGGAACCAAAGCTCTCATGGATGAGGTGGTGAAAGCCACTTCTAGGGGCTGCATCACCATCATAGGTAAGCGGTCCTATACAAAGCTAATACCCATATAAGCTGGCAGAATTCTGATCAGAGGAAGGTGGAATGGAGAACTTCTTCTATGTCTCTTTATTCTGGGTAAATGTTAAGAGGTAAACAGGTAGGTAATTTACAGAGGAGCCTCTTGGTAAGATAGAGTTGGGGGTTTATCAGCTACCTTTTGGGTTGGGGAGCACACTGCCTTACAGTTTTGGTGCCAATCCCTTTTTTTTCTTTTCTCTCTTTTCCCTTTTTACCTGGCTTTCATTCAACAGGTGGTGGAGACACTGCCACTTGCTGTGCCAAATGGAACACGGAGGATAAAGTCAGCCATGTGAGCACTGGGGGTGGTGCCAGTTTGGAGCTCCTGGAAGGGATCCGCCCAAAGGGAGATCCGACTCGTCTGAGGGCGAAGGCGAGACGCGGAAGAGGCCGCAGAGCCGGCAGCAGGCCGCGGGAAGGAAGGTCCGCTGGATTGAGGGCCGAAGGGACGTAGCAGAAGGACGTCCCGCGCAGAATCCAGGTGGCAACACAGGCGAGCAGCCATGGAAAGGACGTCAGCTTCCCCGACAACACCACGGAATTGTCAGTGCCCAACAGCCGAGCCCCTGTCCAGCAGCGGGCAAGGCAGGCGGCGATGAGTTCCGCCGTGGCAATAGGGAGGGGGAAAGCGAAAGTCCCGGAAAGGAGCTGACAGGTGGTGGCAATGCCCCAACCAGTGGGGGTTGCGTCAGCAAACACAGTGCACACCACGCCACGTTGCCTGACAACGGGCCACAACTCCTCATAAAGAGACAGCAACCAGGATTTATACAAGGAGGAGAAAATGAAAGCCATACGGGAAGCAATAGCATGATACAAAGGCATTAAAGCAGCGTATCCACATAGCGTAAAAGGAGCAACATAGTTAAGAATACCAGTCAATCTTTCACAAATTTTGTAATCCAGAGGTTGATTGTCGACGTTTAAACTTACTTGTACAGCTCGTCCATGCCCATCACATCGGTAAAGGCCTTTTGCCACTCCTTGAAGTTGAGCTCGGTCTTGGAGTGCTTGAGCTCCGTCTTACGGAACACGTACATCGGCTGGTTCTTCAGATAGTTAGCCGCCATTGGCTTGGCAAAGGTGTAGGTGGTCCGCGCAGTGCTCCGGTAGCGCTTGCCATTTCCAGTGGTGTAACTCCACTTAAAGGTACTGATGATGGTTTTGTCGTTGGGGTAAGTCTTCTTCGACCTGCACCAGTCCGCAGCGGTCAGCGAGTTGGTCATCACAGGACCGTCAGCAGGGAAACCAGTCCCCTTCACCTGGGCCTCTCCTTTGATGTGGCTTCCCTCGTAGGTGTAGCGGTAGTTAACAGTAAGGGAGGCACCATCTTCAAACTGCATTGTGCGATGGACTTGGTATCCGGAGCCATCTACCATGGCGGCCTGGAAAGGCGACATCCCGTCAGGGTAGGGCAGGTACTGATGGAAGCCATACCCGATATGAGGGACCAGAATCCAGGGGGAGAACTGGAGGTCACCCTTGGTGGACTTCAGGTTTAACTCCTCATAACCATCATTTGGATTGCCGGTGCCCTGACCCACCATGTCAAAGTCCACACCGTTGATGGAGCCAAAGATGTGTAACTCATGTGTCGCTGGGAGAGAGGCCATGTTATCCTCCTCGCCCTTGCTCACCATGGGACCTGGGTTGCTCTCAACATCTCCAGCCAATTTCAAGAGAGCATAATTAGTACACTGGCTAGCAATATTGCTGAGAGCATCCACCCCAGGAAGGACTTTACTAGTACTTTCCTGCCTTTTAGTTCCTGTGCACAGCCCCTAAGTCAACTTAGCATTTTCTGCATCTCCACTTGGCATTAGCTAAAACCTTCCATGTCAAGATTCAGCTAGTGGCCAAGAGATGCAGTGCCAGGAACCCTTAAACAGTTGCACAGCATCTCAGCTCATCTTCACTGCACCCTGGATTTGCATACATTCTTCAAGATCCCATTTGAATTTTTTAGTGACTAAACCATTGTGCATTCTAGAGTGCATATATTTATATTTTGCCTGTTAAAAAGAAAGTGAGCAGTGTTAGCTTAGTTCTCTTTTGATGTAGGTTATTATGATTAGCTTTGTCACTGTTTCACTACTCAGCATGGAAACAAGATGAAATTCCATTTGTAGGTAGTGAGACAAAATTGATGATCCATTAAGTAAACAATAAAAGTGTCCATTGAAACCGTGATTTTTTTTTTTTTCCTGTCATACTTTGTTAGGAAGGGTGAGAATAGAATCTTGAGGAACGGATCAGATGTCTATATTGCTGAATGCAAGAAGTGGGGCAGCAGCAGTGGAGAGATGGGACAATTAGATAAATGTCCATTCTTTATCAAGGGCCTACTTTATGGCAGACATTGTGCTAGTGCTTTTATTCTAACTTTTATTTTTATCAGTTACACATGATCATAATTTAAAAAGTCAAGGCTTATAACAAAAAAGCCCCAGCCCATTCCTCCCATTCAAGATTCCCACTCCCCAGAGGTGACCACTTTCAACTCTTGAGTTTTTCAGGTATATACCTCCATGTTTCTAAGTAATATGCTTATATTGTTCACTTCTTTTTTTTTTATTTTTTAAAGAAATCTATTTCATACCATGGAGGAAGGCTCTGTTCCACATATATTTCCACTTCTTCATTCTCTCGGTATAGTTTTGTCACAATTATAGATTAGATCAAAAGTCTACATAACTAATACAGCTGAGCTATGTAGTATGCTATGATTAAATTTACTTATGTAACTTTTATTGTCTTTGGCATTAACAGTGTTTCAAAAAATTTTCTGTGTATACCCATCAGTGATTCATTCCCAAATCTTCTAGAAGCATAAGTGTCTCAATATATTAAAACATATTGAATAATCCTTGTTAGAGTTATCCCTGCAGGAGTCCTTAGTGCTCCTTTATCCAATTTGTACTTGATGCCCTCTAGGCAGGGTGTACAGCTAGCTGTTGCTCTGGTATTTCCTATAACCTTCTTGGGGATTTCTTTTACCTCCTGTGTTAGACTCCTGTTTTCTGGATTCCCCCTTTTCCCTCTTTCTTGGTCTACTTTTTGTAGAACACAAGACTCTACTAGCTTCCTGAGAAAGGGTGCCTGGGAGGCAAAATCTCTAAGACTTTGTAAGTCTGAAAATGTCTTTATTCGACCCTTATACTTGATTCCTAGTTTGGCTATATATAGAATTTTAGCCTGAGTATCACTTTTTGAGACTCGAAGCCACTGTTTCATTGTCACTATTGAGAATCTAAATGGCCATTCGGATTCTTTCATCATCTTTATAATTTTACCTTCCTATTTCTTTCCTGTCCTTTTAATGGAGTTTTGGGAGAGAGCAGAGGTAAACATGATTTGAAAAAGCCATGTCTGACCAGAAATTCTGTGCTGGAAAGATATGTATTTCACCTTTAGGGACAAAGAAATAAACTTTTGGACAGGACCACAGAGCTAGTAAGTAACAGCAGGGATTCAAGTCCAGGTTTGTCTGGTTCCAGTGGCTGATGCTTTTCCAATGTGCCTCCGTCCCTGACATGATGCTTCTAGGCTATAGATGCTTCTAGACTCTATCCCTGACATGATGCTTCTAGACTATTTTGTTTAACCCTGGATAGAATAGCAAAAGAAAATTTTGGTGGTTGCTCTAAACAAAACAGAAATTTGAAAGCTCAAGTTTTTTCTTCATTTGTATTTTAGTTAATACTGTACCCATATTTGTAGTTAATTTTAAATTGTACCATGTTTCTGCATACCTCTATGGGTACTCAGGAATTCTAGTCCAATTTTTGTGACTTTTTCCTACTGATTACCTTTCCTCCAACGTTTTAAAAATTATTTCAAATGGAACTGAAAGAAGCATAAACTCCCATAAACCCAGCACCTAGACTCTACAATTGCCAACATTATCTATCCAACAATCTCTGACTGTACCTTTTAAGTACTTTCTACTGGACTGTTTCCAGGATCACCCCTCATTTATTTGGGTTGTTAACCTAAAGAATGAATGGGGGAATCTCCAGTCATAAACAACTTGTCAATTAGGCAAATATTTGAGTTCCTTCTATGTGCTTAAAGACGTGATAGAGGGAATATAAGAGCATTTATGTTCTGAAGGAATTTTTAACCTAACCAAAGAAGATAGTAGATAACTTGTGTGCATATAAGCTAGAACAGTATAAGGGGCCGGGCATGGTGGCTTACGCCTGTAATCCCAGGACTTTGGGAGGCCAAGGCGGGCAGATCACCTGTCAGGAGT |
| Forward plasmid backbone insertion | TGCAGCCCTGAGTTCTGGTCTTGGTGGAGGTGGTGTTTAAGTAGCTTTTCTTGATAGCTCATCTTCTCTTTCACCTCTACCCCTCAGGGCTTGGACTGTGGTCCTGAAAGCAGCAAGAAGTATGCTGAGGCTGTCACTCGGGCTAAGCAGATTGTGTGGAATGGTCCTGTGGGGGTATTTGAATGGGAAGCTTTTGCCCGGGGAACCAAAGCTCTCATGGATGAGGTGGTGAAAGCCACTTCTAGGGGCTGCATCACCATCATAGGTAAGCGGTCCTATACAAAGCTAATACCCATATAAGCTGGCAGAATTCTGATCAGAGGAAGGTGGAATGGAGAACTTCTTCTATGTCTCTTTATTCTGGGTAAATGTTAAGAGGTAAACAGGTAGGTAATTTACAGAGGAGCCTCTTGGTAAGATAGAGTTGGGGGTTTATCAGCTACCTTTTGGGTTGGGGAGCACACTGCCTTACAGTTTTGGTGCCAATCCCTTTTTTTTCTTTTCTCTCTTTTCCCTTTTTACCTGGCTTTCATTCAACAGGTGGTGGAGACACTGCCACTTGCTGTGCCAAATGGAACACGGAGGATAAAGTCAGCCATGTGAGCACTGGGGGTGGTGCCAGTTTGGAGCTCCTGGAAGGTGAGGGTCTTCTGTTTTTTGGCTTGTTTGGGATAAGGGTGGACTGTGCAGTGAGAGGTGGGTACAGGTGGCACTTTTCGGGGAAATGTGCGCGGAACCCCTATTTGTTTATTTTTCTAAATACATTCAAATATGTATCCGCTCATGAGACAATAACCCTGATAAATGCTTCAATAATATTGAAAAAGGAAGAGTATGAGTATTCAACATTTCCGTGTCGCCCTTATTCCCTTTTTTGCGGCATTTTGCCTTCCTGTTTTTGCTCACCCAGAAACGCTGGTGAAAGTAAAAGATGCTGAAGATCAGTTGGGTGCACGAGTGGGTTACATCGAACTGGATCTCAACAGCGGTAAGATCCTTGAGAGTTTTCGCCCCGAAGAACGTTTTCCAATGATGAGCACTTTTAAAGTTCTGCTATGTGGCGCGGTATTATCCCGTATTGACGCCGGGCAAGAGCAACTCGGTCGCCGCATACACTATTCTCAGAATGACTTGGTTGAGTACTCACCAGTCACAGAAAAGCATCTTACGGATGGCATGACAGTAAGAGAATTATGCAGTGCTGCCATAACCATGAGTGATAACACTGCGGCCAACTTACTTCTGACAACGATCGGAGGACCGAAGGAGCTAACCGCTTTTTTGCACAACATGGGGGATCATGTAACTCGCCTTGATCGTTGGGAACCGGAGCTGAATGAAGCCATACCAAACGACGAGCGTGACACCACGATGCCTGTAGCAATGGCAACAACGTTGCGCAAACTATTAACTGGCGAACTACTTACTCTAGCTTCCCGGCAACAATTAATAGACTGGATGGAGGCGGATAAAGTTGCAGGACCACTTCTGCGCTCGGCCCTTCCGGCTGGCTGGTTTATTGCTGATAAATCTGGAGCCGGTGAGCGTGGGTCTCGCGGTATCATTGCAGCACTGGGGCCAGATGGTAAGCCCTCCCGTATCGTAGTTATCTACACGACGGGGAGTCAGGCAACTATGGATGAACGAAATAGACAGATCGCTGAGATAGGTGCCTCACTGATTAAGCATTGGTAACTGTCAGACCAAGTTTACTCATATATACTTTAGATTGATTTAAAACTTCATTTTTAATTTAAAAGGATCTAGGTGAAGATCCTTTTTGATAATCTCATGACCAAAATCCCTTAACGTGAGTTTTCGTTCCACTGAGCGTCAGACCCCGTAGAAAAGATCAAAGGATCTTCTTGAGATCCTTTTTTTCTGCGCGTAATCTGCTGCTTGCAAACAAAAAAACCACCGCTACCAGCGGTGGTTTGTTTGCCGGATCAAGAGCTACCAACTCTTTTTCCGAAGGTAACTGGCTTCAGCAGAGCGCAGATACCAAATACTGTCCTTCTAGTGTAGCCGTAGTTAGGCCACCACTTCAAGAACTCTGTAGCACCGCCTACATACCTCGCTCTGCTAATCCTGTTACCAGTGGCTGCTGCCAGTGGCGATAAGTCGTGTCTTACCGGGTTGGACTCAAGACGATAGTTACCGGATAAGGCGCAGCGGTCGGGCTGAACGGGGGGTTCGTGCACACAGCCCAGCTTGGAGCGAACGACCTACACCGAACTGAGATACCTACAGCGTGAGCTATGAGAAAGCGCCACGCTTCCCGAAGGGAGAAAGGCGGACAGGTATCCGGTAAGCGGCAGGGTCGGAACAGGAGAGCGCACGAGGGAGCTTCCAGGGGGAAACGCCTGGTATCTTTATAGTCCTGTCGGGTTTCGCCACCTCTGACTTGAGCGTCGATTTTTGTGATGCTCGTCAGGGGGGCGGAGCCTATGGAAAAACGCCAGCAACGCGGCCTTTTTACGGTTCCTGGCCTTTTGCTGGCCTTTTGCTCACATGTTCTTTCCTGCGTTATCCCCTGATTCTGTGGATAACCGTATTACCGCCTTTGAGTGAGCTGATACCGCTCGCCGCAGCCGAACGACCGAGCGCAGCGAGTCAGTGAGCGAGGAAGCGGAAGAGCGCCCAATACGCAAACCGCCTCTCCCCGCGCGTTGGCCGATTCATTAATGCAGCTGGCACGACAGGTTTCCCGACTGGAAAGCGGGCAGTGAGCGCAACGCAATTAATGTGAGTTAGCTCACTCATTAGGCACCCCAGGCTTTACACTTTATGCTTCCGGCTCGTATGTTGTGTGGAATTGTGAGCGGATAACAATTTCACACAGGAGAATGGAGTGGAGAAAGTTAGAAGGTAGTGTTGTCATTAGCAGTCATTACTACCTGGGCAGTACAGAGGAACTTCAGATAAAGCTCCTGGCATCCACTGAGGCGGGGAGGGACAGATAGAAACTTGGTCTGAGAGTTATGGTCTAGTAGACCTGGAATCCACAATGTAAAAGTTGGCCAGCTCCTGGCCATATATCCTAAAAAAGAGCTGGCATGTTATTGGGAAGATAAAGTGGGGGAAATCTGGCTTACTGGGCCCTATAGTAATGCTGTCTATGTATGTGTGCTCTCTCAAAAACAGGTAAAGTCCTTCCTGGGGTGGATGCTCTCAGCAATATTTAGTACTTTCCTGCCTTTTAGTTCCTGTGCACAGCCCCTAAGTCAACTTAGCATTTTCTGCATCTCCACTTGGCATTAGCTAAAACCTTCCATGTCAAGATTCAGCTAGTGGCCAAGAGATGCAGTGCCAGGAACCCTTAAACAGTTGCACAGCATCTCAGCTCATCTTCACTGCACCCTGGATTTGCATACATTCTTCAAGATCCCATTTGAATTTTTTAGTGACTAAACCATTGTGCATTCTAGAGTGCATATATTTATATTTTGCCTGTTAAAAAGAAAGTGAGCAGTGTTAGCTTAGTTCTCTTTTGATGTAGGTTATTATGATTAGCTTTGTCACTGTTTCACTACTCAGCATGGAAACAAGATGAAATTCCATTTGTAGGTAGTGAGACAAAATTGATGATCCATTAAGTAAACAATAAAAGTGTCCATTGAAACCGTGATTTTTTTTTTTTTCCTGTCATACTTTGTTAGGAAGGGTGAGAATAGAATCTTGAGGAACGGATCAGATGTCTATATTGCTGAATGCAAGAAGTGGGGCAGCAGCAGTGGAGAGATGGGACAATTAGATAAATGTCCATTCTTTATCAAGGGCCTACTTTATGGCAGACATTGTGCTAGTGCTTTTATTCTAACTTTTATTTTTATCAGTTACACATGATCATAATTTAAAAAGTCAAGGCTTATAACAAAAAAGCCCCAGCCCATTCCTCCCATTCAAGATTCCCACTCCCCAGAGGTGACCACTTTCAACTCTTGAGTTTTTCAGGTATATACCTCCATGTTTCTAAGTAATATGCTTATATTGTTCACTTCTTTTTTTTTTATTTTTTAAAGAAATCTATTTCATACCATGGAGGAAGGCTCTGTTCCACATATATTTCCACTTCTTCATTCTCTCGGTATAGTTTTGTCACAATTATAGATTAGATCAAAAGTCTACATAACTAATACAGCTGAGCTATGTAGTATGCTATGATTAAATTTACTTATGTAACTTTTATTGTCTTTGGCATTAACAGTGTTTCAAAAAATTTTCTGTGTATACCCATCAGTGATTCATTCCCAAATCTTCTAGAAGCATAAGTGTCTCAATATATTAAAACATATTGAATAATCCTTGTTAGAGTTATCCCTGCAGGAGTCCTTAGTGCTCCTTTATCCAATTTGTACTTGATGCCCTCTAGGCAGGGTGTACAGCTAGCTGTTGCTCTGGTATTTCCTATAACCTTCTTGGGGATTTCTTTTACCTCCTGTGTTAGACTCCTGTTTTCTGGATTCCCCCTTTTCCCTCTTTCTTGGTCTACTTTTTGTAGAACACAAGACTCTACTAGCTTCCTGAGAAAGGGTGCCTGGGAGGCAAAATCTCTAAGACTTTGTAAGTCTGAAAATGTCTTTATTCGACCCTTATACTTGATTCCTAGTTTGGCTATATATAGAATTTTAGCCTGAGTATCACTTTTTGAGACTCGAAGCCACTGTTTCATTGTCACTATTGAGAATCTAAATGGCCATTCGGATTCTTTCATCATCTTTATAATTTTACCTTCCTATTTCTTTCCTGTCCTTTTAATGGAGTTTTGGGAGAGAGCAGAGGTAAACATGATTTGAAAAAGCCATGTCTGACCAGAAATTCTGTGCTGGAAAGATATGTATTTCACCTTTAGGGACAAAGAAATAAACTTTTGGACAGGACCACAGAGCTAGTAAGTAACAGCAGGGATTCAAGTCCAGGTTTGTCTGGTTCCAGTGGCTGATGCTTTTCCAATGTGCCTCCGTCCCTGACATGATGCTTCTAGGCTATAGATGCTTCTAGACTCTATCCCTGACATGATGCTTCTAGACTATTTTGTTTAACCCTGGATAGAATAGCAAAAGAAAATTTTGGTGGTTGCTCTAAACAAAACAGAAATTTGAAAGCTCAAGTTTTTTCTTCATTTGTATTTTAGTTAATACTGTACCCATATTTGTAGTTAATTTTAAATTGTACCATGTTTCTGCATACCTCTATGGGTACTCAGGAATTCTAGTCCAATTTTTGTGACTTTTTCCTACTGATTACCTTTCCTCCAACGTTTTAAAAATTATTTCAAATGGAACTGAAAGAAGCATAAACTCCCATAAACCCAGCACCTAGACTCTACAATTGCCAACATTATCTATCCAACAATCTCTGACTGTACCTTTTAAGTACTTTCTACTGGACTGTTTCCAGGATCACCCCTCATTTATTTGGGTTGTTAACCTAAAGAATGAATGGGGGAATCTCCAGTCATAAACAACTTGTCAATTAGGCAAATATTTGAGTTCCTTCTATGTGCTTAAAGACGTGATAGAGGGAATATAAGAGCATTTATGTTCTGAAGGAATTTTTAACCTAACCAAAGAAGATAGTAGATAACTTGTGTGCATATAAGCTAGAACAGTATAAGGGGCCGGGCATGGTGGCTTACGCCTGTAATCCCAGGACTTTGGGAGGCCAAGGCGGGCAGATCACCTGTCAGGAGT |
| Reverse plasmid backbone insertion | TGCAGCCCTGAGTTCTGGTCTTGGTGGAGGTGGTGTTTAAGTAGCTTTTCTTGATAGCTCATCTTCTCTTTCACCTCTACCCCTCAGGGCTTGGACTGTGGTCCTGAAAGCAGCAAGAAGTATGCTGAGGCTGTCACTCGGGCTAAGCAGATTGTGTGGAATGGTCCTGTGGGGGTATTTGAATGGGAAGCTTTTGCCCGGGGAACCAAAGCTCTCATGGATGAGGTGGTGAAAGCCACTTCTAGGGGCTGCATCACCATCATAGGTAAGCGGTCCTATACAAAGCTAATACCCATATAAGCTGGCAGAATTCTGATCAGAGGAAGGTGGAATGGAGAACTTCTTCTATGTCTCTTTATTCTGGGTAAATGTTAAGAGGTAAACAGGTAGGTAATTTACAGAGGAGCCTCTTGGTAAGATAGAGTTGGGGGTTTATCAGCTACCTTTTGGGTTGGGGAGCACACTGCCTTACAGTTTTGGTGCCAATCCCTTTTTTTTCTTTTCTCTCTTTTCCCTTTTTACCTGGCTTTCATTCAACAGGTGGTGGAGACACTGCCACTTGCTGTGCCAAATGGAACACGGAGGATAAAGTCAGCCATGTGAGCACTGGGGGTGGTGCCAGTTTGGAGCTCCTGGAAGGTGAGGGTCTTCTGTTTTTTGGCTTGTTTGGGATAAGGGTGGACTGTGCAGTGAGAGGTGGGTATCCTGTGTGAAATTGTTATCCGCTCACAATTCCACACAACATACGAGCCGGAAGCATAAAGTGTAAAGCCTGGGGTGCCTAATGAGTGAGCTAACTCACATTAATTGCGTTGCGCTCACTGCCCGCTTTCCAGTCGGGAAACCTGTCGTGCCAGCTGCATTAATGAATCGGCCAACGCGCGGGGAGAGGCGGTTTGCGTATTGGGCGCTCTTCCGCTTCCTCGCTCACTGACTCGCTGCGCTCGGTCGTTCGGCTGCGGCGAGCGGTATCAGCTCACTCAAAGGCGGTAATACGGTTATCCACAGAATCAGGGGATAACGCAGGAAAGAACATGTGAGCAAAAGGCCAGCAAAAGGCCAGGAACCGTAAAAAGGCCGCGTTGCTGGCGTTTTTCCATAGGCTCCGCCCCCCTGACGAGCATCACAAAAATCGACGCTCAAGTCAGAGGTGGCGAAACCCGACAGGACTATAAAGATACCAGGCGTTTCCCCCTGGAAGCTCCCTCGTGCGCTCTCCTGTTCCGACCCTGCCGCTTACCGGATACCTGTCCGCCTTTCTCCCTTCGGGAAGCGTGGCGCTTTCTCATAGCTCACGCTGTAGGTATCTCAGTTCGGTGTAGGTCGTTCGCTCCAAGCTGGGCTGTGTGCACGAACCCCCCGTTCAGCCCGACCGCTGCGCCTTATCCGGTAACTATCGTCTTGAGTCCAACCCGGTAAGACACGACTTATCGCCACTGGCAGCAGCCACTGGTAACAGGATTAGCAGAGCGAGGTATGTAGGCGGTGCTACAGAGTTCTTGAAGTGGTGGCCTAACTACGGCTACACTAGAAGGACAGTATTTGGTATCTGCGCTCTGCTGAAGCCAGTTACCTTCGGAAAAAGAGTTGGTAGCTCTTGATCCGGCAAACAAACCACCGCTGGTAGCGGTGGTTTTTTTGTTTGCAAGCAGCAGATTACGCGCAGAAAAAAAGGATCTCAAGAAGATCCTTTGATCTTTTCTACGGGGTCTGACGCTCAGTGGAACGAAAACTCACGTTAAGGGATTTTGGTCATGAGATTATCAAAAAGGATCTTCACCTAGATCCTTTTAAATTAAAAATGAAGTTTTAAATCAATCTAAAGTATATATGAGTAAACTTGGTCTGACAGTTACCAATGCTTAATCAGTGAGGCACCTATCTCAGCGATCTGTCTATTTCGTTCATCCATAGTTGCCTGACTCCCCGTCGTGTAGATAACTACGATACGGGAGGGCTTACCATCTGGCCCCAGTGCTGCAATGATACCGCGAGACCCACGCTCACCGGCTCCAGATTTATCAGCAATAAACCAGCCAGCCGGAAGGGCCGAGCGCAGAAGTGGTCCTGCAACTTTATCCGCCTCCATCCAGTCTATTAATTGTTGCCGGGAAGCTAGAGTAAGTAGTTCGCCAGTTAATAGTTTGCGCAACGTTGTTGCCATTGCTACAGGCATCGTGGTGTCACGCTCGTCGTTTGGTATGGCTTCATTCAGCTCCGGTTCCCAACGATCAAGGCGAGTTACATGATCCCCCATGTTGTGCAAAAAAGCGGTTAGCTCCTTCGGTCCTCCGATCGTTGTCAGAAGTAAGTTGGCCGCAGTGTTATCACTCATGGTTATGGCAGCACTGCATAATTCTCTTACTGTCATGCCATCCGTAAGATGCTTTTCTGTGACTGGTGAGTACTCAACCAAGTCATTCTGAGAATAGTGTATGCGGCGACCGAGTTGCTCTTGCCCGGCGTCAATACGGGATAATACCGCGCCACATAGCAGAACTTTAAAAGTGCTCATCATTGGAAAACGTTCTTCGGGGCGAAAACTCTCAAGGATCTTACCGCTGTTGAGATCCAGTTCGATGTAACCCACTCGTGCACCCAACTGATCTTCAGCATCTTTTACTTTCACCAGCGTTTCTGGGTGAGCAAAAACAGGAAGGCAAAATGCCGCAAAAAAGGGAATAAGGGCGACACGGAAATGTTGAATACTCATACTCTTCCTTTTTCAATATTATTGAAGCATTTATCAGGGTTATTGTCTCATGAGCGGATACATATTTGAATGTATTTAGAAAAATAAACAAATAGGGGTTCCGCGCACATTTCCCCGAAAAGTGCCACCTGGAATGGAGTGGAGAAAGTTAGAAGGTAGTGTTGTCATTAGCAGTCATTACTACCTGGGCAGTACAGAGGAACTTCAGATAAAGCTCCTGGCATCCACTGAGGCGGGGAGGGACAGATAGAAACTTGGTCTGAGAGTTATGGTCTAGTAGACCTGGAATCCACAATGTAAAAGTTGGCCAGCTCCTGGCCATATATCCTAAAAAAGAGCTGGCATGTTATTGGGAAGATAAAGTGGGGGAAATCTGGCTTACTGGGCCCTATAGTAATGCTGTCTATGTATGTGTGCTCTCTCAAAAACAGGTAAAGTCCTTCCTGGGGTGGATGCTCTCAGCAATATTTAGTACTTTCCTGCCTTTTAGTTCCTGTGCACAGCCCCTAAGTCAACTTAGCATTTTCTGCATCTCCACTTGGCATTAGCTAAAACCTTCCATGTCAAGATTCAGCTAGTGGCCAAGAGATGCAGTGCCAGGAACCCTTAAACAGTTGCACAGCATCTCAGCTCATCTTCACTGCACCCTGGATTTGCATACATTCTTCAAGATCCCATTTGAATTTTTTAGTGACTAAACCATTGTGCATTCTAGAGTGCATATATTTATATTTTGCCTGTTAAAAAGAAAGTGAGCAGTGTTAGCTTAGTTCTCTTTTGATGTAGGTTATTATGATTAGCTTTGTCACTGTTTCACTACTCAGCATGGAAACAAGATGAAATTCCATTTGTAGGTAGTGAGACAAAATTGATGATCCATTAAGTAAACAATAAAAGTGTCCATTGAAACCGTGATTTTTTTTTTTTTCCTGTCATACTTTGTTAGGAAGGGTGAGAATAGAATCTTGAGGAACGGATCAGATGTCTATATTGCTGAATGCAAGAAGTGGGGCAGCAGCAGTGGAGAGATGGGACAATTAGATAAATGTCCATTCTTTATCAAGGGCCTACTTTATGGCAGACATTGTGCTAGTGCTTTTATTCTAACTTTTATTTTTATCAGTTACACATGATCATAATTTAAAAAGTCAAGGCTTATAACAAAAAAGCCCCAGCCCATTCCTCCCATTCAAGATTCCCACTCCCCAGAGGTGACCACTTTCAACTCTTGAGTTTTTCAGGTATATACCTCCATGTTTCTAAGTAATATGCTTATATTGTTCACTTCTTTTTTTTTTATTTTTTAAAGAAATCTATTTCATACCATGGAGGAAGGCTCTGTTCCACATATATTTCCACTTCTTCATTCTCTCGGTATAGTTTTGTCACAATTATAGATTAGATCAAAAGTCTACATAACTAATACAGCTGAGCTATGTAGTATGCTATGATTAAATTTACTTATGTAACTTTTATTGTCTTTGGCATTAACAGTGTTTCAAAAAATTTTCTGTGTATACCCATCAGTGATTCATTCCCAAATCTTCTAGAAGCATAAGTGTCTCAATATATTAAAACATATTGAATAATCCTTGTTAGAGTTATCCCTGCAGGAGTCCTTAGTGCTCCTTTATCCAATTTGTACTTGATGCCCTCTAGGCAGGGTGTACAGCTAGCTGTTGCTCTGGTATTTCCTATAACCTTCTTGGGGATTTCTTTTACCTCCTGTGTTAGACTCCTGTTTTCTGGATTCCCCCTTTTCCCTCTTTCTTGGTCTACTTTTTGTAGAACACAAGACTCTACTAGCTTCCTGAGAAAGGGTGCCTGGGAGGCAAAATCTCTAAGACTTTGTAAGTCTGAAAATGTCTTTATTCGACCCTTATACTTGATTCCTAGTTTGGCTATATATAGAATTTTAGCCTGAGTATCACTTTTTGAGACTCGAAGCCACTGTTTCATTGTCACTATTGAGAATCTAAATGGCCATTCGGATTCTTTCATCATCTTTATAATTTTACCTTCCTATTTCTTTCCTGTCCTTTTAATGGAGTTTTGGGAGAGAGCAGAGGTAAACATGATTTGAAAAAGCCATGTCTGACCAGAAATTCTGTGCTGGAAAGATATGTATTTCACCTTTAGGGACAAAGAAATAAACTTTTGGACAGGACCACAGAGCTAGTAAGTAACAGCAGGGATTCAAGTCCAGGTTTGTCTGGTTCCAGTGGCTGATGCTTTTCCAATGTGCCTCCGTCCCTGACATGATGCTTCTAGGCTATAGATGCTTCTAGACTCTATCCCTGACATGATGCTTCTAGACTATTTTGTTTAACCCTGGATAGAATAGCAAAAGAAAATTTTGGTGGTTGCTCTAAACAAAACAGAAATTTGAAAGCTCAAGTTTTTTCTTCATTTGTATTTTAGTTAATACTGTACCCATATTTGTAGTTAATTTTAAATTGTACCATGTTTCTGCATACCTCTATGGGTACTCAGGAATTCTAGTCCAATTTTTGTGACTTTTTCCTACTGATTACCTTTCCTCCAACGTTTTAAAAATTATTTCAAATGGAACTGAAAGAAGCATAAACTCCCATAAACCCAGCACCTAGACTCTACAATTGCCAACATTATCTATCCAACAATCTCTGACTGTACCTTTTAAGTACTTTCTACTGGACTGTTTCCAGGATCACCCCTCATTTATTTGGGTTGTTAACCTAAAGAATGAATGGGGGAATCTCCAGTCATAAACAACTTGTCAATTAGGCAAATATTTGAGTTCCTTCTATGTGCTTAAAGACGTGATAGAGGGAATATAAGAGCATTTATGTTCTGAAGGAATTTTTAACCTAACCAAAGAAGATAGTAGATAACTTGTGTGCATATAAGCTAGAACAGTATAAGGGGCCGGGCATGGTGGCTTACGCCTGTAATCCCAGGACTTTGGGAGGCCAAGGCGGGCAGATCACCTGTCAGGAGT |
