# Supplementary material for: GREPore-seq: A Robust Workflow to Detect Changes After Gene Editing Through Long-range PCR and Nanopore Sequencing
Source: Genomics Proteomics Bioinformatics. 2022 Jun 23;21(6):1221–36. doi: 10.1016/j.gpb.2022.06.002 (PMC11082256; doi:10.1016/j.gpb.2022.06.002)
Supplement: Supplementary Table S3 — Guide RNA sequences [file mmc3.docx]

**Table S3 Guide RNA sequences**

| **Site** | **Guide RNA sequences** |
| --- | --- |
| *AAVS1* | TAAGGAATCTGCCTAACAGG |
| *B2M* | AGTCACATGGTTCACACGGC |
| *BCL11A-1/3* | CTAACAGTTGCTTTTATCAC |
| *BCL11A-2/4* | GTAGGCGACCAACATGGGGT |
| *EEF2* | CTTCCTGGACAAATTGTAGG |
| *TRAC* | TGTGCTAGACATGAGGTCTA |
| *TRBC* | GGAGAATGACGAGTGGACCC |
| *PGK1* | GTGCAGTGAGAGGTGGGTAGAA |
