# Supplementary material for: GREPore-seq: A Robust Workflow to Detect Changes After Gene Editing Through Long-range PCR and Nanopore Sequencing
Source: Genomics Proteomics Bioinformatics. 2022 Jun 23;21(6):1221–36. doi: 10.1016/j.gpb.2022.06.002 (PMC11082256; doi:10.1016/j.gpb.2022.06.002)
Supplement: Supplementary Table S4 — Forward primers with/without barcodes and the reverse primer for nanopore sequencing data [file mmc4.docx]

**Table S4 Forward primers with/without barcodes and the reverse primer for nanopore sequencing data**

*Note*: red letters represent individual barcodes.

*AAVS1 (long-range 3928 bp)*

| **ID** | **Primer sequences (5'-3')** |
| --- | --- |
| AAVS1-F-BC1 | aagaggagTGCAAACAGGAAGTGAACGG |
| AAVS1-F-BC2 | ctggaaatTGCAAACAGGAAGTGAACGG |
| AAVS1-F-BC3 | cttgttggTGCAAACAGGAAGTGAACGG |
| AAVS1-F-BC4 | tatgcgttTGCAAACAGGAAGTGAACGG |
| AAVS1-F-BC5 | tgttcaagTGCAAACAGGAAGTGAACGG |
| AAVS1-F-BC6 | aaactttgTGCAAACAGGAAGTGAACGG |
| AAVS1-F-BC7 | ggaccgatTGCAAACAGGAAGTGAACGG |
| AAVS1-F-BC8 | aattatccTGCAAACAGGAAGTGAACGG |
| AAVS1-F-BC9 | aaacaaacTGCAAACAGGAAGTGAACGG |
| AAVS1-F-BC10 | accgcctaTGCAAACAGGAAGTGAACGG |
| AAVS1-F-BC11 | ccaggttcTGCAAACAGGAAGTGAACGG |
| AAVS1-F-BC12 | cctaacgcTGCAAACAGGAAGTGAACGG |
| AAVS1-F-BC13 | cgctcttcTGCAAACAGGAAGTGAACGG |
| AAVS1-F-BC14 | gggtaacgTGCAAACAGGAAGTGAACGG |
| AAVS1-F-BC15 | tatacgacTGCAAACAGGAAGTGAACGG |
| AAVS1-F-BC16 | aacggactTGCAAACAGGAAGTGAACGG |
| AAVS1-F-BC17 | gacaacgcTGCAAACAGGAAGTGAACGG |
| AAVS1-F-BC18 | ttagtccgTGCAAACAGGAAGTGAACGG |
| AAVS1-F-BC19 | attccggaTGCAAACAGGAAGTGAACGG |
| AAVS1-F-BC20 | ccgacatcTGCAAACAGGAAGTGAACGG |
| AAVS1-F-BC21 | accgtttaTGCAAACAGGAAGTGAACGG |
| AAVS1-F-BC22 | gggtaacgatTGCAAACAGGAAGTGAACGG |
| AAVS1-F-BC23 | tatacgactaTGCAAACAGGAAGTGAACGG |
| AAVS1-F-BC24 | aacggactagTGCAAACAGGAAGTGAACGG |
| AAVS1-F-BC25 | gacaacgcaaTGCAAACAGGAAGTGAACGG |
| AAVS1-F-BC26 | atgataactaggTGCAAACAGGAAGTGAACGG |
| AAVS1-F-BC27 | catctcatctcgTGCAAACAGGAAGTGAACGG |
| AAVS1-F-BC28 | cctgaagacgttTGCAAACAGGAAGTGAACGG |
| AAVS1-F-BC29 | gcagaagtctatTGCAAACAGGAAGTGAACGG |
| AAVS1-F-BC30 | gttcagtaagacTGCAAACAGGAAGTGAACGG |
| AAVS1-F-BC31 | taccagtcaatcTGCAAACAGGAAGTGAACGG |
| AAVS1-F-BC32 | tgctcatctgctTGCAAACAGGAAGTGAACGG |
| AAVS1-F-BC33 | tgttggaacttcTGCAAACAGGAAGTGAACGG |
| AAVS1-F-BC34 | aggagacatcagTGCAAACAGGAAGTGAACGG |
| AAVS1-F-BC35 | aacgactatgtcTGCAAACAGGAAGTGAACGG |
| AAVS1-F-BC36 | gtagctgattccTGCAAACAGGAAGTGAACGG |
| AAVS1-F-BC37 | taagacactcgtTGCAAACAGGAAGTGAACGG |
| AAVS1-F-BC38 | gatacatatcgaTGCAAACAGGAAGTGAACGG |
| AAVS1-F-BC39 | ttctggtgactgTGCAAACAGGAAGTGAACGG |
| AAVS1-F-BC40 | agttggaggttcTGCAAACAGGAAGTGAACGG |
| AAVS1-F-BC41 | actcgtcgtctaTGCAAACAGGAAGTGAACGG |
| AAVS1-Reverse | CGACCTACTCTCTTCCGCAT |

*BCL11A-3 (long-range 3863 bp)*

| **ID** | **Primer sequences (5’-3’)** |
| --- | --- |
| BCL11A-3-F-BC1 | aagaggagGTGTGGTGTTCGGAGTCCTA |
| BCL11A-3-F-BC2 | ctggaaatGTGTGGTGTTCGGAGTCCTA |
| BCL11A-3-F-BC3 | cttgttggGTGTGGTGTTCGGAGTCCTA |
| BCL11A-3-F-BC4 | tatgcgttGTGTGGTGTTCGGAGTCCTA |
| BCL11A-3-F-BC5 | tgttcaagGTGTGGTGTTCGGAGTCCTA |
| BCL11A-3-F-BC6 | aaactttgGTGTGGTGTTCGGAGTCCTA |
| BCL11A-3-F-BC7 | ggaccgatGTGTGGTGTTCGGAGTCCTA |
| BCL11A-3-F-BC8 | aattatccGTGTGGTGTTCGGAGTCCTA |
| BCL11A-3-F-BC9 | aaacaaacGTGTGGTGTTCGGAGTCCTA |
| BCL11A-3-F-BC10 | accgcctaGTGTGGTGTTCGGAGTCCTA |
| BCL11A-3-F-BC11 | ccaggttcGTGTGGTGTTCGGAGTCCTA |
| BCL11A-3-F-BC12 | cctaacgcGTGTGGTGTTCGGAGTCCTA |
| BCL11A-3-F-BC13 | cgctcttcGTGTGGTGTTCGGAGTCCTA |
| BCL11A-3-F-BC14 | ttagtccgGTGTGGTGTTCGGAGTCCTA |
| BCL11A-3-F-BC15 | gacaacgcGTGTGGTGTTCGGAGTCCTA |
| BCL11A-3-F-BC16 | gggtaacgGTGTGGTGTTCGGAGTCCTA |
| BCL11A-3-F-BC17 | tatacgacGTGTGGTGTTCGGAGTCCTA |
| BCL11A-3-F-BC18 | aacggactGTGTGGTGTTCGGAGTCCTA |
| BCL11A-3-F-BC19 | agactcgtGTGTGGTGTTCGGAGTCCTA |
| BCL11A-3-F-BC20 | attccggaGTGTGGTGTTCGGAGTCCTA |
| BCL11A-3-F-BC21 | actcgagaGTGTGGTGTTCGGAGTCCTA |
| BCL11A-3-F-BC22 | ttagtccgacGTGTGGTGTTCGGAGTCCTA |
| BCL11A-3-F-BC23 | gacaacgcttGTGTGGTGTTCGGAGTCCTA |
| BCL11A-3-F-BC24 | gggtaacgtaGTGTGGTGTTCGGAGTCCTA |
| BCL11A-3-F-BC25 | tatacgacatGTGTGGTGTTCGGAGTCCTA |
| BCL11A-3-F-BC26 | acgccacgttGTGTGGTGTTCGGAGTCCTA |
| BCL11A-3-F-BC27 | cactctcaggGTGTGGTGTTCGGAGTCCTA |
| BCL11A-3-F-BC28 | cagtgaccagGTGTGGTGTTCGGAGTCCTA |
| BCL11A-3-F-BC29 | ccaggctcttGTGTGGTGTTCGGAGTCCTA |
| BCL11A-3-F-BC30 | agattactcaGTGTGGTGTTCGGAGTCCTA |
| BCL11A-3-F-BC31 | cgactagaccGTGTGGTGTTCGGAGTCCTA |
| BCL11A-3- Reverse | AGGAGCGGCAGTTTAAGTCT |

*BCL11A-4 (long-range 5313 bp)*

| **ID** | **Primer sequences (5’-3’)** |
| --- | --- |
| BCL11A-4-F-BC1 | aagaggagGCTGTGCTTTCTTCTATGATTCCTC |
| BCL11A-4-F-BC2 | ctggaaatGCTGTGCTTTCTTCTATGATTCCTC |
| BCL11A-4-F-BC3 | cttgttggGCTGTGCTTTCTTCTATGATTCCTC |
| BCL11A-4-F-BC4 | tatgcgttGCTGTGCTTTCTTCTATGATTCCTC |
| BCL11A-4-F-BC5 | tgttcaagGCTGTGCTTTCTTCTATGATTCCTC |
| BCL11A-4-F-BC6 | aaactttgGCTGTGCTTTCTTCTATGATTCCTC |
| BCL11A-4-F-BC7 | ggaccgatGCTGTGCTTTCTTCTATGATTCCTC |
| BCL11A-4-F-BC8 | aattatccGCTGTGCTTTCTTCTATGATTCCTC |
| BCL11A-4-F-BC9 | aaacaaacGCTGTGCTTTCTTCTATGATTCCTC |
| BCL11A-4-F-BC10 | accgcctaGCTGTGCTTTCTTCTATGATTCCTC |
| BCL11A-4-F-BC11 | ccaggttcGCTGTGCTTTCTTCTATGATTCCTC |
| BCL11A-4-F-BC12 | cctaacgcGCTGTGCTTTCTTCTATGATTCCTC |
| BCL11A-4-F-BC13 | cgctcttcGCTGTGCTTTCTTCTATGATTCCTC |
| BCL11A-4-F-BC14 | gatctgacagGCTGTGCTTTCTTCTATGATTCCTC |
| BCL11A-4-F-BC15 | gcaactgtctGCTGTGCTTTCTTCTATGATTCCTC |
| BCL11A-4-F-BC16 | gctgatcggaGCTGTGCTTTCTTCTATGATTCCTC |
| BCL11A-4-F-BC17 | gacgactaagGCTGTGCTTTCTTCTATGATTCCTC |
| BCL11A-4-F-BC18 | gtccggtgaaGCTGTGCTTTCTTCTATGATTCCTC |
| BCL11A-4-F-BC19 | gtgcatatccGCTGTGCTTTCTTCTATGATTCCTC |
| BCL11A-4-F-BC20 | taaggaatccGCTGTGCTTTCTTCTATGATTCCTC |
| BCL11A-4-F-BC21 | tatgctatgcGCTGTGCTTTCTTCTATGATTCCTC |
| BCL11A-4-F-BC22 | tcgcgacactGCTGTGCTTTCTTCTATGATTCCTC |
| BCL11A-4-F-BC23 | ttcggatggtGCTGTGCTTTCTTCTATGATTCCTC |
| BCL11A-4-Reverse | TGAAATCTCCCTTCTTTACGGTTCT |

*EEF2 (long-range 5287 bp)*

| **ID** | **Primer sequences (5’-3’)** |
| --- | --- |
| EEF2-F-BC1 | atgataactaggAAGTCTTGGGCTCCTCAGTC |
| EEF2-F-BC2 | catctcatctcgAAGTCTTGGGCTCCTCAGTC |
| EEF2-F-BC3 | cctgaagacgttAAGTCTTGGGCTCCTCAGTC |
| EEF2-F-BC4 | gcagaagtctatAAGTCTTGGGCTCCTCAGTC |
| EEF2-F-BC5 | gttcagtaagacAAGTCTTGGGCTCCTCAGTC |
| EEF2-F-BC6 | taccagtcaatcAAGTCTTGGGCTCCTCAGTC |
| EEF2-F-BC7 | tgctcatctgctAAGTCTTGGGCTCCTCAGTC |
| EEF2-F-BC8 | tgttggaacttcAAGTCTTGGGCTCCTCAGTC |
| EEF2-F-BC9 | aggagacatcagAAGTCTTGGGCTCCTCAGTC |
| EEF2-F-BC10 | aacgactatgtcAAGTCTTGGGCTCCTCAGTC |
| EEF2-F-BC11 | gtagctgattccAAGTCTTGGGCTCCTCAGTC |
| EEF2-F-BC12 | taagacactcgtAAGTCTTGGGCTCCTCAGTC |
| EEF2-F-BC13 | gatacatatcgaAAGTCTTGGGCTCCTCAGTC |
| EEF2-F-BC14 | ttctggtgactgAAGTCTTGGGCTCCTCAGTC |
| EEF2-F-BC15 | agttggaggttcAAGTCTTGGGCTCCTCAGTC |
| EEF2-F-BC16 | actcgtcgtctaAAGTCTTGGGCTCCTCAGTC |
| EEF2-F-BC17 | aaagatccAAGTCTTGGGCTCCTCAGTC |
| EEF2-F-BC18 | aatatgctAAGTCTTGGGCTCCTCAGTC |
| EEF2-F-BC19 | actcgagtAAGTCTTGGGCTCCTCAGTC |
| EEF2-F-BC20 | agatgatgAAGTCTTGGGCTCCTCAGTC |
| EEF2-F-BC21 | attgtacgAAGTCTTGGGCTCCTCAGTC |
| EEF2-F-BC22 | ccactgacAAGTCTTGGGCTCCTCAGTC |
| EEF2-F-BC23 | gatcttacAAGTCTTGGGCTCCTCAGTC |
| EEF2-F-BC24 | ggagcgatAAGTCTTGGGCTCCTCAGTC |
| EEF2-F-BC25 | acgccacgttAAGTCTTGGGCTCCTCAGTC |
| EEF2-F-BC26 | cactctcaggAAGTCTTGGGCTCCTCAGTC |
| EEF2-F-BC27 | cagtgaccagAAGTCTTGGGCTCCTCAGTC |
| EEF2-F-BC28 | ccaggctcttAAGTCTTGGGCTCCTCAGTC |
| EEF2-F-BC29 | agattactctAAGTCTTGGGCTCCTCAGTC |
| EEF2-F-BC30 | cgactagaccAAGTCTTGGGCTCCTCAGTC |
| EEF2-F-BC31 | gatctgacagAAGTCTTGGGCTCCTCAGTC |
| EEF2-F-BC32 | gcaactgtctAAGTCTTGGGCTCCTCAGTC |
| EEF2-Reverse | GACCAACCAGGCCAAGCAAA |

*PGK1 (long-range 3552 bp)*

| **ID** | **Primer sequences (5’-3’)** |
| --- | --- |
| PGK1-F-BC1 | aagactgagaTGCAGCCCTGAGTTCTGGTC |
| PGK1-F-BC2 | aatggtaacgTGCAGCCCTGAGTTCTGGTC |
| PGK1-F-BC3 | acagttatgcTGCAGCCCTGAGTTCTGGTC |
| PGK1-F-BC4 | accgcaagacTGCAGCCCTGAGTTCTGGTC |
| PGK1-F-BC5 | acgcctcttaTGCAGCCCTGAGTTCTGGTC |
| PGK1-F-BC6 | agctaccatgTGCAGCCCTGAGTTCTGGTC |
| PGK1-F-BC7 | atcttggagtTGCAGCCCTGAGTTCTGGTC |
| PGK1-F-BC8 | ccggtagttcTGCAGCCCTGAGTTCTGGTC |
| PGK1-F-BC9 | cgagaaggaaTGCAGCCCTGAGTTCTGGTC |
| PGK1-F-BC10 | cggacacctaTGCAGCCCTGAGTTCTGGTC |
| PGK1-F-BC11 | gcgtcgtgaaTGCAGCCCTGAGTTCTGGTC |
| PGK1-F-BC12 | gtagaatcctTGCAGCCCTGAGTTCTGGTC |
| PGK1-F-BC13 | gtccacagctTGCAGCCCTGAGTTCTGGTC |
| PGK1-F-BC14 | gttactcggtTGCAGCCCTGAGTTCTGGTC |
| PGK1-F-BC15 | aacaacaaccTGCAGCCCTGAGTTCTGGTC |
| PGK1-F-BC16 | aacaaggtggTGCAGCCCTGAGTTCTGGTC |
| PGK1-F-BC17 | aaccgattccTGCAGCCCTGAGTTCTGGTC |
| PGK1-F-BC18 | aactctcgccTGCAGCCCTGAGTTCTGGTC |
| PGK1-F-BC19 | agtgtggtccTGCAGCCCTGAGTTCTGGTC |
| PGK1-F-BC20 | ctctatacacTGCAGCCCTGAGTTCTGGTC |
| PGK1-F-BC21 | tggtgcataaTGCAGCCCTGAGTTCTGGTC |
| PGK1-F-BC22 | ttcgatgcggTGCAGCCCTGAGTTCTGGTC |
| PGK1-F-BC23 | ttgtcttctgTGCAGCCCTGAGTTCTGGTC |
| PGK1-F-BC24 | tccttaatccTGCAGCCCTGAGTTCTGGTC |
| PGK1-F-BC25 | aacggtggTGCAGCCCTGAGTTCTGGTC |
| PGK1-F-BC26 | accttcttTGCAGCCCTGAGTTCTGGTC |
| PGK1-F-BC27 | attgagccTGCAGCCCTGAGTTCTGGTC |
| PGK1-F-BC28 | ccaggaagTGCAGCCCTGAGTTCTGGTC |
| PGK1-F-BC29 | cgtccaatTGCAGCCCTGAGTTCTGGTC |
| PGK1-F-BC30 | gtgtcgttTGCAGCCCTGAGTTCTGGTC |
| PGK1-F-BC31 | agccactcTGCAGCCCTGAGTTCTGGTC |
| PGK1-F-BC32 | cagtatagTGCAGCCCTGAGTTCTGGTC |
| PGK1-F-BC33 | catccaccTGCAGCCCTGAGTTCTGGTC |
| PGK1-F-BC34 | cgacaacgTGCAGCCCTGAGTTCTGGTC |
| PGK1-F-BC35 | ctctgacaTGCAGCCCTGAGTTCTGGTC |
| PGK1-F-BC36 | gcagcactTGCAGCCCTGAGTTCTGGTC |
| PGK1-F-BC37 | aactaattgcggTGCAGCCCTGAGTTCTGGTC |
| PGK1-F-BC38 | aagttgcgctaaTGCAGCCCTGAGTTCTGGTC |
| PGK1-F-BC39 | acattaacctcgTGCAGCCCTGAGTTCTGGTC |
| PGK1-F-BC40 | acgatagttgtgTGCAGCCCTGAGTTCTGGTC |
| PGK1-F-BC41 | agcggatgtcacTGCAGCCCTGAGTTCTGGTC |
| PGK1-F-BC42 | agtatgaagccaTGCAGCCCTGAGTTCTGGTC |
| PGK1-F-BC43 | atgcacctctgtTGCAGCCCTGAGTTCTGGTC |
| PGK1-F-BC44 | caatacgctgcaTGCAGCCCTGAGTTCTGGTC |
| PGK1-F-BC45 | cgattacacaagTGCAGCCCTGAGTTCTGGTC |
| PGK1-F-BC46 | ctagtgttcaagTGCAGCCCTGAGTTCTGGTC |
| PGK1-F-BC47 | cttattgagacgTGCAGCCCTGAGTTCTGGTC |
| PGK1-F-BC48 | tagtgacgatggTGCAGCCCTGAGTTCTGGTC |
| PGK1-F-BC49 | accgcacctgttatTGCAGCCCTGAGTTCTGGTC |
| PGK1-F-BC50 | acgagccaatcctaTGCAGCCCTGAGTTCTGGTC |
| PGK1-F-BC51 | actactcctgacctTGCAGCCCTGAGTTCTGGTC |
| PGK1-F-BC52 | agaacaatgagaggTGCAGCCCTGAGTTCTGGTC |
| PGK1-F-BC53 | agagatgtcgttggTGCAGCCCTGAGTTCTGGTC |
| PGK1-F-BC54 | cacttaggacttctTGCAGCCCTGAGTTCTGGTC |
| PGK1-F-BC55 | cataggaccgcgtaTGCAGCCCTGAGTTCTGGTC |
| PGK1-F-BC56 | catgactggtgttgTGCAGCCCTGAGTTCTGGTC |
| PGK1-F-BC57 | cgaaccgatcatgcTGCAGCCCTGAGTTCTGGTC |
| PGK1-F-BC58 | cgtaagttcgaccgTGCAGCCCTGAGTTCTGGTC |
| PGK1-F-BC59 | gagcgcaatagccaTGCAGCCCTGAGTTCTGGTC |
| PGK1-F-BC60 | tacatacgtccaagTGCAGCCCTGAGTTCTGGTC |

| **Site name** | **Product size (bp)** | **Primer-F sequences**  **(5’-3’)** | **Primer-R sequences**  **(5’-3’)** |
| --- | --- | --- | --- |
| *B2M* | 5666 | ACTGTGCCTCTTACTTTCGGTTTTG | TGTCACCCCAACTATGCCATTTAAC |
| *BCL11A-1* | 8159 | CATACAATAGAGGCATTTGGAACCC | AGGAGCGGCAGTTTAAGTCT |
| *BCL11A-2* | 8443 | TTACAACACAATGAAAGGGTAAGGC | TGAAATCTCCCTTCTTTACGGTTCT |
| *TRAC* | 6485 | TAATAGAGACACGGGGCATGGTATG | TAGGAGCAAATGTACCCTGGAGTTC |
| *TRBC* | 5093 | AACCGGTACCATTTGTAGTTAGGCT | CTTCCTCAACTAACTTCGACATGGC |
