# Supplementary material for: GREPore-seq: A Robust Workflow to Detect Changes After Gene Editing Through Long-range PCR and Nanopore Sequencing
Source: Genomics Proteomics Bioinformatics. 2022 Jun 23;21(6):1221–36. doi: 10.1016/j.gpb.2022.06.002 (PMC11082256; doi:10.1016/j.gpb.2022.06.002)
Supplement: Supplementary Table S5 — Forward primers with barcodes and the reverse primer for Illumina paired-end 150 bp sequencing data [file mmc5.docx]

**Table S5 Forward primers with barcodes and the reverse primer for Illumina paired-end 150 bp sequencing data**

| **ID** | **Primer sequences (5'-3')** |
| --- | --- |
| EEF2-F-BC1 | tcacgCAGTCTCCAGGTGTCGTCTG |
| EEF2-F-BC2 | gatgtCAGTCTCCAGGTGTCGTCTG |
| EEF2-F-BC3 | taggcCAGTCTCCAGGTGTCGTCTG |
| EEF2-F-BC4 | gaccaCAGTCTCCAGGTGTCGTCTG |
| EEF2-F-BC5 | cagtgCAGTCTCCAGGTGTCGTCTG |
| EEF2-F-BC6 | ccaatCAGTCTCCAGGTGTCGTCTG |
| EEF2-F-BC7 | agatcCAGTCTCCAGGTGTCGTCTG |
| EEF2-F-BC8 | cttgaCAGTCTCCAGGTGTCGTCTG |
| EEF2-F-BC9 | agttgCAGTCTCCAGGTGTCGTCTG |
| EEF2-F-BC10 | gcataCAGTCTCCAGGTGTCGTCTG |
| EEF2-F-BC11 | ctatcCAGTCTCCAGGTGTCGTCTG |
| EEF2-F-BC12 | actcgCAGTCTCCAGGTGTCGTCTG |
| EEF2-F-BC13 | ggcatCAGTCTCCAGGTGTCGTCTG |
| EEF2-F-BC14 | gtcttCAGTCTCCAGGTGTCGTCTG |
| EEF2-F-BC15 | tgactCAGTCTCCAGGTGTCGTCTG |
| EEF2-F-BC16 | ttattCAGTCTCCAGGTGTCGTCTG |
| EEF2- Reverse | GTTTGACCACTGGCAGATCC |

*Note*: red letters represent individual barcodes.
